# Acyclovir Has Low but Detectable Influence on HLA-B*57:01 Specificity without Inducing Hypersensitivity

**DOI:** 10.1371/journal.pone.0124878

**Published:** 2015-05-29

**Authors:** Imir G. Metushi, Amanda Wriston, Priyanka Banerjee, Bjoern Oliver Gohlke, A. Michelle English, Andrew Lucas, Carrie Moore, John Sidney, Soren Buus, David A. Ostrov, Simon Mallal, Elizabeth Phillips, Jeffrey Shabanowitz, Donald F. Hunt, Robert Preissner, Bjoern Peters

**Affiliations:** 1 Division of Vaccine Discovery, La Jolla Institute for Allergy and Immunology, La Jolla, California, United States of America; 2 Department of Chemistry, University of Virginia, Charlottesville, Virginia, United States of America; 3 Charite—University Medicine Berlin, Institute of Physiology & Experimental Clinical Research Center, Berlin, Germany; 4 Graduate School of Computational Systems Biology, Humboldt-Universität zu Berlin, Berlin, Germany; 5 German Cancer Consortium (DKTK), Heidelberg, Germany; 6 Institute for Immunology and Infectious Diseases, Murdoch University, Murdoch, Western Australia, Australia; 7 Laboratory of Experimental Immunology, Faculty of Health Sciences, University of Copenhagen, Copenhagen, Denmark; 8 Department of Pathology, Immunology and Laboratory Medicine, University of Florida College of Medicine, Gainesville, Florida, United States of America; 9 Vanderbilt University School of Medicine, Nashville, Tennessee, United States of America; 10 Department of Pathology, University of Virginia, Charlottesville, Virginia, United States of America; Johns Hopkins University, UNITED STATES

## Abstract

Immune mediated adverse drug reactions (IM-ADRs) remain a significant source of patient morbidity that have more recently been shown to be associated with specific class I and/or II human leukocyte antigen (HLA) alleles. Abacavir-induced hypersensitivity syndrome is a CD8^+^ T cell dependent IM-ADR that is exclusively mediated by HLA-B*57:01. We and others have previously shown that abacavir can occupy the floor of the peptide binding groove of HLA-B*57:01 molecules, increasing the affinity of certain self peptides resulting in an altered peptide-binding repertoire. Here, we have identified another drug, acyclovir, which appears to act in a similar fashion. As with abacavir, acyclovir showed a dose dependent increase in affinity for peptides with valine and isoleucine at their C-terminus. In agreement with the binding studies, HLA-B*57:01 peptide-elution studies performed in the presence of acyclovir revealed an increased number of endogenously bound peptides with a C-terminal isoleucine. Accordingly, we have hypothesized that acyclovir acts by the same mechanism as abacavir, although our data also suggest the overall effect is much smaller: the largest changes of peptide affinity for acyclovir were 2-5 fold, whereas for abacavir this effect was as much as 1000-fold. Unlike abacavir, acyclovir is not known to cause IM-ADRs. We conclude that the modest effect of acyclovir on HLA binding affinity in contrast to the large effect of abacavir is insufficient to trigger a hypersensitivity syndrome. We further support this by functional *in vitro* studies where acyclovir, unlike abacavir, was unable to produce an increase in IFN-γ upon expansion of HLA-B*57:01^+^ PBMCs from healthy donors. Using abacavir and acyclovir as examples we therefore propose an in vitro pre-clinical screening strategy, whereby thresholds can be applied to MHC-peptide binding assays to determine the likelihood that a drug could cause a clinically relevant IM-ADR.

## Introduction

Adverse drug reactions (ADRs) represent a significant health problem, and add significantly to the cost of drug development. Immune mediated adverse drug reactions (IM-ADRs) are amongst the most difficult to predict. Recently, an increasing number of studies have implicated specific human leukocyte antigen (HLA) alleles with various IM-ADRs [1,2]. This is exemplified by the strong association between the nucleoside reverse transcriptase inhibitor abacavir and a drug hypersensitivity syndrome in individuals carrying HLA-B*57:01, with a 55% positive predictive value and 100% negative predictive value [3]. Our group and others have proposed the altered repertoire hypothesis to explain abacavir-induced hypersensitivity [4–6]. In an earlier study, we were able to illustrate that abacavir interacts with residues in the F-pocket of HLA-B*57:01 which are typically in contact with the side chain of the C-terminal residue of the bound peptide. This results in an alteration of the specificity of HLA-B*57:01 for self-peptides, and a corresponding presentation of de-novo self-peptides with much higher affinities in the presence of the drug [4].

The assays performed to study the effect of abacavir on HLA binding could be utilized to screen other drugs for potential effects on the self-peptide repertoire. This would provide a potential *in vitro* screening strategy to identify drugs at highest risk of causing IM-ADRs *in vivo* when used in human donors that express the affected HLA molecules. Such an *in vitro* screen would be of high value in the early phases of drug development as a typical phase I or II clinical trial would be underpowered to ensure that every known (or even every common) HLA molecule is represented at sufficient frequency, particularly taking into account the incomplete positive predictive value of any HLA allele for a specific IM-ADR. Thus severe HLA associated IM-ADRs are often only detected when a drug comes into broad use and significant investment has already been made.

We have established two *in vitro* assays that are potentially suited to screen whether a drug can alter the repertoire of HLA-bound self-peptides. The first is a purified HLA binding assay, which can be used together with a positional scanning combinatorial peptide libraries (PSCPL) approach to measure changes in the peptide-binding specificity in the presence or absence of the drug in question. The second is a peptide elution assay performed on single HLA allele transfected cell lines performed in the presence and absence of the drug directly identifying the effects on the self-peptide repertoire [4]. For these assays to become useful in practice, it is necessary to establish what distinguishes a safe drug from a drug that can induce a treatment-limiting IM-ADR. In this study, we have used *in silico* approaches to identify drugs on the market that have structural similarities to abacavir and performed *in vitro* binding and elution experiments to test whether these drugs can alter the repertoire of HLA-B*57:01 in a manner similar to abacavir. By comparing the safety profile of these drugs with the detected effect on HLA binding, we propose a strategy to identify and test a preliminary threshold for effects on HLA binding that can be considered safe.

## Materials and Methods

### Ethics statement

This study was approved by the Australian Bone Marrow Donor Registry as the source of healthy donor HLA-B*57:01 positive and control PBMCs. In addition the use of human cells was approved by the ethics committee of the Institute for Immunology and Infectious Diseases, Murdoch University, Australia. The use of HLA-B*57:01 and B*58:01 cell lines was as previously described by our group (see reference 4).

### Database Searching

**Fig 1:**
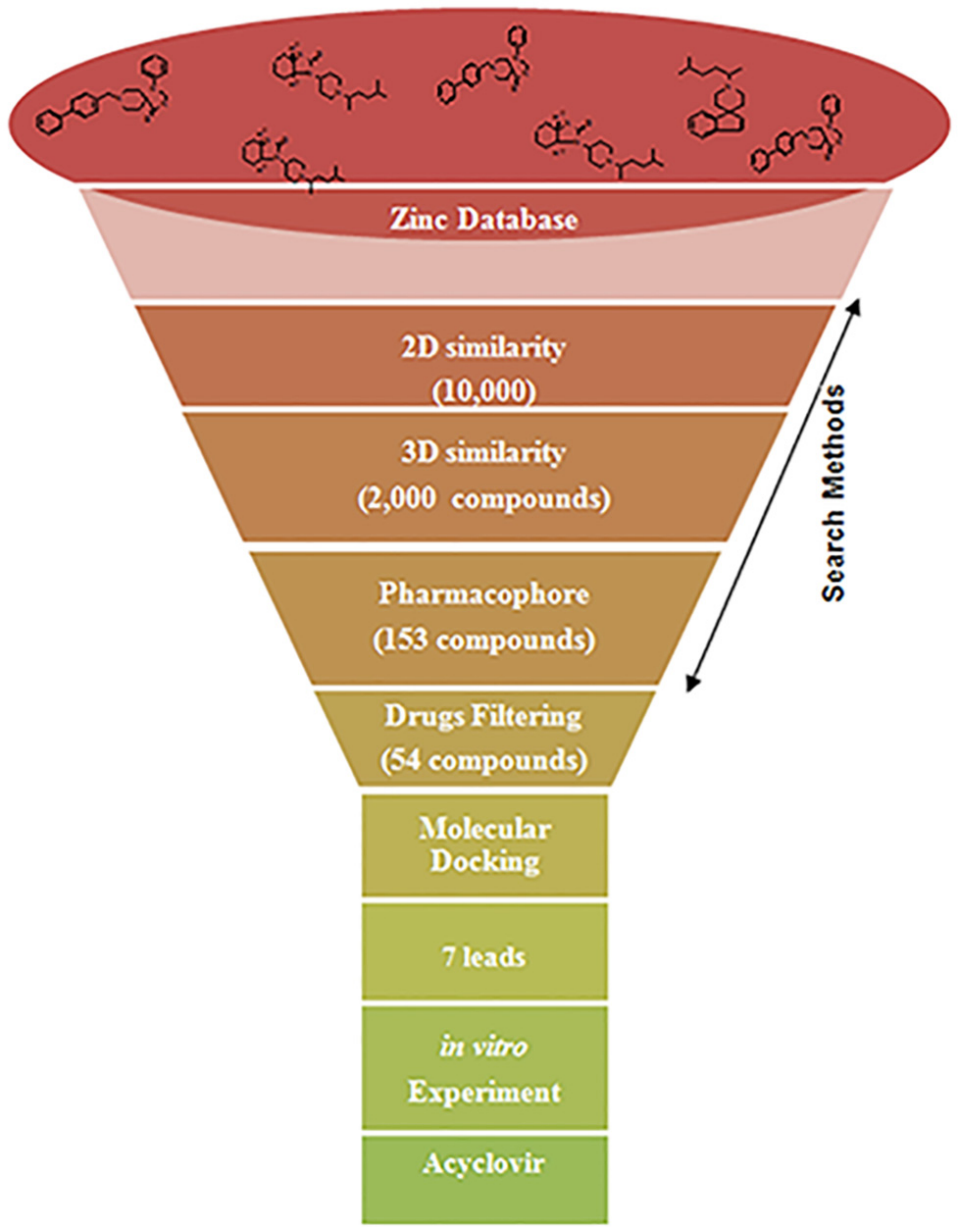
The workflow of the virtual screening protocol for screening of similar drugs to abacavir.

The screening of molecules is based on the assumption that 'structurally similar molecules might have similar biological property' [7]. A screening protocol was built containing a series of filters to carry out the virtual screening using 2D and 3D similarity, taking into account the location of pharmacophoric groups (Fig 1). Virtual screening of the ZINC database [8], a free database of commercially-available compounds, was carried out as described below. The 2D structure of the reference molecule abacavir was screened against the ZINC database using a concatenated fingerprint consisting of the 'FP2' and 'FP4' fingerprints of Mychem (http://mychem.sourceforge.net/). The degree of structural similarity was calculated using a Tanimoto score[9], considering a threshold of 0.60 and above.

Additionally, 3D similarity-based screening was carried out for approximately 10,000 molecules obtained from 2D-based similarity screening results using a superimposition algorithm developed previously [10]. The 3D similarity score was calculated in terms of root mean square deviation (rmsd), by optimization of an objective function that takes into account the distance between the atoms. This score reflects the quality of the superposition between the query molecule and the database entries. Using a threshold of rmsd 1.5 Å, around 2,000 compounds were scored, and for each compound up to 100 computed conformers were considered for the comparison. Furthermore, a special preferred screening condition was defined in order to retain certain pharmacophoric features obtained from known structure activity relationship (SAR). In previous docking studies [4], it was observed that the purine core of abacavir is important for binding. Accordingly, the purine core was marked as one of the chemical features to be retained in the screened molecules. A similar criterion was established for the hydroxyl group of abacavir, which is also hypothesized to be involved in binding. Moreover, the spatial arrangement of the aromatic ring is assumed to be an important recognition element. On the basis of these filtering criteria, a total of 153 compounds were investigated further. Finally, with reference to the chemical tractability and the quality of superimposition, 54 compounds were selected for docking studies.

### Molecular Docking

**Fig 2:**
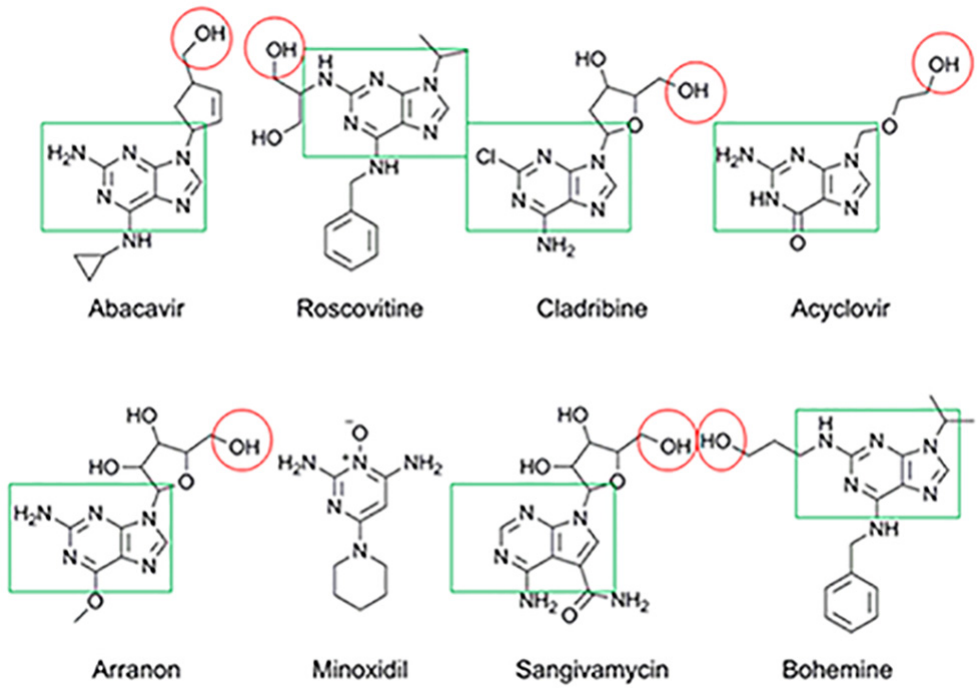
Chemical structures for abacavir and the other seven drugs that were predicted to bind in the F-pocket of HLA-B*57:01.

To further filter the hit list, docking studies were carried out using GOLD 5.2 [11] and the GOLDScore scoring function. In the present study, the abacavir-induced MHC complex (PDB ID: 3UPR) was selected as the input structure for docking studies. Residues around the original ligand (radius 10.0 Å) were defined as the active site, which also covered the peptide binding area of the MHC molecule. Docking studies of the previously selected 54 compounds were performed using the standard parameters and further inspected visually using PyMOL (The PyMOL Molecular Graphics System, Version 1.5.0.4 Schrödinger, LLC.). Based on reasonable binding conformations, seven candidates were forwarded as leads for experimental validation (Fig 2). The analysis of the docking results is provided in (S2 & S2 Fig; with reference to abacavir and acyclovir).

### Drugs

Roscovitine, cladribine, acyclovir, arranon, minoxidil, sangivamycin and bohemine were purchased from Fisher Scientific (Pitsburgh, PA). Abacavir sulfate was purchased from LGM Pharma (Nashville, TN). Each drug was initially re-suspended at 30 mg/mL in 50% dimethylsulfoxide (DMSO) in phosphate buffered saline (PBS, pH 7.2). The drugs were then suspended in the final assay at a concentration of 2, 1 or 0.5 mg/mL containing 3.3% DMSO in PBS (denoted as vehicle).

### HLA Binding Assays

Quantitative assays to measure the binding affinity of peptides to purified HLA-B*57:01 molecules based on the inhibition of binding of a radiolabeled standard peptide were performed as previously described [12]. Briefly, 1–10 nM of radiolabeled peptide was co-incubated at room temperature with 1 nM to 1 μM purified HLA-B*57:01 in the presence of 1–3 μM human β2-microglobulin (Scripps Laboratories; San Diego, CA) and a mixture of protease inhibitors. After a 2-day incubation, binding of the radiolabeled peptide to the MHC class I molecule was determined by capturing MHC/peptide complexes on Greiner Lumitrac 600 microplates (Greiner Bio-One; Monroe, North Carilina) coated with the W6/32 antibody, and measuring bound counts per minute (cpm) using the TopCount microscintillation gamma counter (PerkinElmer; Waltham, Massachusetts). For competition assays, the concentration of peptide yielding IC_50_ of the binding of the radiolabeled peptide was calculated. Peptides were typically tested at six different concentrations covering a 100,000-fold dose range.

For direct binding studies in the absence of a competitor peptide, a purified peptide with sequence KAAKYRVSV was ordered from Synthetic Biomolecules (San Diego, CA) and its affinity for either HLA-B*57:01 or HLA-B*58:01 was tested in the presence or absence of abacavir, or acyclovir, in a dose dependent manner.

### Peptide Elution and Identification

For elution studies, 4–5 x 10^9^ cells from the HLA-B*57:01 single allele transfected 721.221 cell line were used [13]. Treated cells were incubated with acyclovir at a concentration of 500 μg/mL for 14 hr at 37°. Cells were harvested and stored at -80°C. Upon thawing, cells were lysed using cell lysis buffer in the presence of protease inhibitors. The lysate was centrifuged at 100,000 x g for 1 hr and the supernatant was collected and passed through a 0.8/0.2 μm filter (VWR International, TX). The filtrate was collected and passed through a sepharose CL-4B (Sigma-Aldrich, MO) column to capture any remaining materials in the lysate that may clog the antibody columns. The filtrate was then passaged through a column packed with protein A sepharose (PAS) beads (sigma) coated with MK-D6 (MTCC HB-3, VA) antibody which served to as an irrelevant antibody (specific for the mouse class II molecule, I-A^d^) used to derive a negative control peptide extract. Next, the filtrate was passed through a second PAS column coated with W6/32 antibody (MTCC HB-95) which captures HLA class I molecules. The columns were washed sequentially with: 1) 2 column volumes of lysis buffer, 2) 20 column volumes of 20 mM Tris-HCL (pH 8.0, 150 mM NaCl), 3) 20 column volumes of 20 mM Tri-HCl (pH 8.0, 1.0 M NaCl), and 4) 20 column volumes of 20 mM Tris-HCl (pH 8.0), and then eluted with 4 column volumes of 0.2 M glacial acetic acid. The eluted peptides were then collected and spun at 3,500g at 4°C until 98% of the solution had passed through Millipore ultrafiltration units with a 3,500 Da cut-off (EMD Millipore, MA) to exclude β-2 microglobulin. The filtrate was then collected and concentrated in vacuo for LC-MS analysis.

Peptides were analyzed by nanoflow high-performance liquid chromatography (HPLC)/microelectrospray ionization, coupled directly to a Thermo Fisher Scientific FT-ICR mass spectrometer, equipped with a home-built, front-end electron transfer dissociation (FETD) source [14]. An aliquot of peptide sample was taken to dryness in a vacuum concentrator. Peptides were amidated in a solution that contained 1M histamine-HCl (20 μL) in 1M pyridine-HCl buffer and 0.1M EDC in pyridine-HCl buffer [15]. The solution was sonicated for 3 hours before being dried in a vacuum concentrator and then reconstituted in 0.1% acetic acid.

Data were acquired as previously described [16]. In brief, a pre-column, loaded with 5×10^7^ to 2×10^8^ cell equivalents of HLA-B*57:01 eluted peptides was connected with polytetrafluoroethylene tubing (0.06-in. o.d. and 0.012-in. i.d.; Zeus Industrial Products) to the end of an analytical HPLC column (360 μm o.d.×50 μm i.d.) containing 6–7 cm of C_18_ reverse-phase packing material (5-μm particles; YMC). Peptides were eluted through a laser-pulled electrospray tip directly into the mass spectrometer with an Agilent 1100 series binary LC pump at a flow rate of ~60 nL/min. Elution gradients utilized were as follows: solvent A was 0.1 M acetic acid in H_2_O and solvent B was 70% acetonitrile. For the analyses, collisionally activated dissociation (CAD) and ETD fragmentation were performed sequentially on the same parent ions. The FETD reagent was azulene and the ion-ion reaction time was set to 45 ms. The instrument was operated in a data-dependent mode where a full-scan mass spectrum was acquired with the high resolution mass analyzer and this was then followed by sequential acquisition of CAD and/ or ETD MS/MS spectra in the linear trap on the top 5 or 10 most abundant, non-excluded ions observed in the full-scan spectrum.

Data from MS/MS experiments were searched against the Swiss-Prot [17] human database using the Open Mass Spectrometry Search Algorithm software [18] to generate a list of candidate peptide sequences. Instrument parameters included a precursor mass tolerance of ±0.01 Da and a monoisotopic fragment ion mass tolerance of ±0.35 Da. Database search parameters allowed variable modifications for phosphorylation on serine, threonine, and tyrosine residues and oxidation of methionine. In addition for the histamine reaction data the variable modification for histamine to aspartic acid, glutamic acid, and the C-terminus were included. Database assignments of drug-specific peptides were confirmed by manual interpretation of the corresponding MS/MS spectra.

### Culture and Detection of Drug Responsive T Cells

Drug responsive short-term T cell lines were produced from cryopreserved PBMC, as previously described [19]. Briefly, HLA-B*57:01 positive PBMC samples were cultured in the presence or absence of 10 μg/mL abacavir (pure substance; GSK) or 10 μg/mL acyclovir (Sigma-Aldrich) for 14 days. From day 3 and every 3 days, 50% of media was replaced and 20 U/ml of recombinant human interleukin (IL)-2 (Peprotech) was added to cultures. To enumerate drug responsive T cells, C1R.B57 antigen presenting cells (APC) were cultured overnight with or without 10 μg/mL abacavir, or 10 μg/mL acyclovir, and then washed. Treated or untreated C1R.B57 APC were added at a 1:10 ratio to PBMC and incubated at 37°C/5% CO2 for six hours, in the presence of 20 μg/mL Brefeldin-A (Sigma-Aldrich) for the final 4 hours. Samples were surface labeled for CD4 and CD8 expression on ice and then fixed and permeabilized for the detection of intra-cellular IFN-γ, as previously described [19]. Abacavir responsive cells were then detected using flow cytometry (Gallios; Beckman Coulter). Lymphocytes were gated on the basis of forward and side scatter properties. The lymphocyte gate was then gated for CD8, but not CD4, positivity using a Boolean logic gate. The frequency of CD8 and IFN-γ double positive cells was determined using Kaluza software (Beckman Coulter).

### Statistical Analysis

Statistical analyses were performed using GraphPad Prism (GraphPad Software, San Diego, CA). Data were analyzed using column statistics, t-test, Mann-Whitney U-test or one-sided Fisher’s exact test. All p-values < 0.05 were considered significant.

## Results

### Computational Identification of Drugs Predicted to have a Similar Effect to Abacavir on HLA-B*57:01 Binding

We set out to identify drugs currently in use that are structurally similar to abacavir and therefore could show a similar effect on HLA-B*57:01 binding. The screening process was initiated by filtering approximately 3.5 million entries from the ZINC database using a 2D similarity-based method (Fig 1). Since the chemical space was huge, a strict threshold of 0.60 Tanimoto score was considered. The subset was further reduced using 3D similarity and presence of the pharmacophoric groups as additional filtering criteria. Based on the degree of superimposition and chemical tractability, the enriched subset was further analyzed through molecular docking and visual inspection.

Previous analysis[4] of the abacavir-exposed HLA-B*57:01 complex suggested that interactions of residues Ser166, Asp114, Ile124 and Tyr74 of the MHC molecule and the purine core of abacavir play a crucial role in the molecular recognition. Hence, this information was utilized for pose selection from the molecular docking studies.

Visual inspection was carried out based on the pre-analyzed receptor-ligand interactions of abacavir-induced MHC complex, maximum chemical similarity between the reference structure and the database entries and, lastly, the occupancy of the ligand in the active site defined. This led to the identification of seven drugs as candidates for further analysis (Fig 2).

### *In Vitro* Binding Studies to Identify Peptides that Bind with High Affinity to HLA-B*57:01 in the Presence of Acyclovir

**Fig 3:**
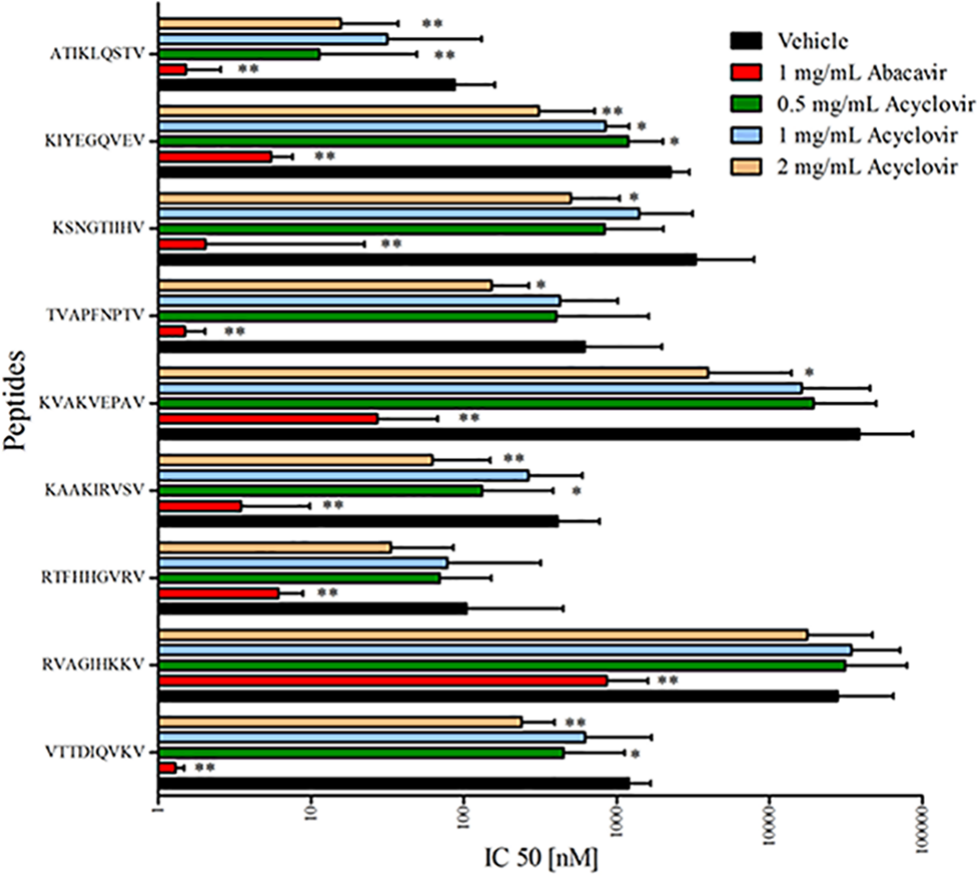
Effects of abacavir and acyclovir on HLA-B*57:01 binding specificity. Specific peptides with a terminal valine that showed an increased affinity for HLA-B*57:01 in the presence of abacavir were tested. Values are represented as geometric mean with 95% CI of two independent runs in triplicates, analyzed for statistical significance by Mann-Whitney U test comparing log IC_50_ values vs. vehicle; p < 0.05 was considered significant (*p < 0.05; **p < 0.01; ***p < 0.001).

Next, we tested if the presence of each drug could enhance the HLA-B*57:01 binding affinity for three 9-mer peptides with a C-terminal valine known to bind HLA-B*57:01 with high affinity only in the presence of abacavir. This screening indicated that acyclovir lead to a small but consistent increase in the HLA-B*57:01 affinity of these three peptides (S3 Fig), while the other compounds had a smaller or no effect at all. We then screened a larger number of 9-mer peptides ending with V in dose dependent concentration of acyclovir and also used abacavir as a positive control. As expected from our previous studies [4], the affinity of HLA-B*57:01 for each of these peptides increase significantly in the presence of abacavir (Fig 3). For most of the peptides, acyclovir had a qualitatively similar, albeit much smaller, effect (Fig 3).

### Positional scanning combinatorial library analysis

Since acyclovir was predicted to be similar to abacavir in terms of its capacity to interact with HLA-B*57:01, we next determined if the effect of acyclovir could have been detected when screening a set of 9-mer PSCPL systematically scanning the effect of each naturally occurring amino acid at each position in a 9-mer peptide. This approach was successful in identifying that abacavir primarily impacts the interaction between the peptide C-terminal residue and the HLA-B*57:01 molecule [4].

**Fig 4:**
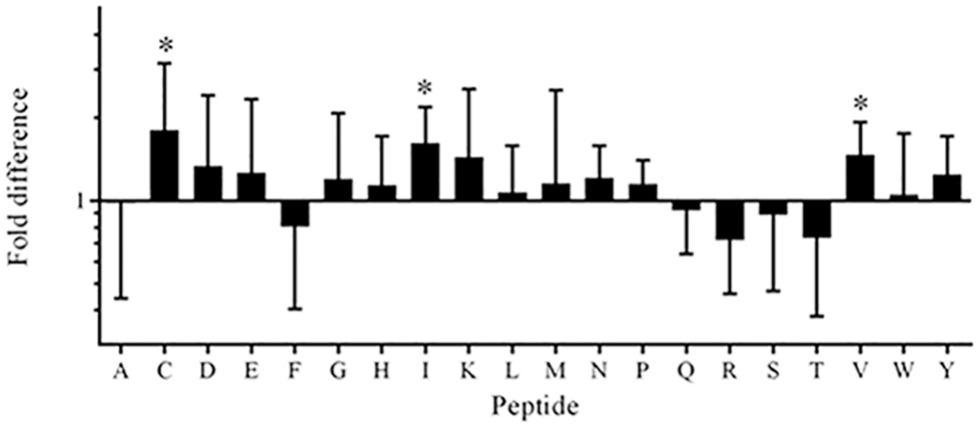
Effects of acyclovir (2 mg/mL) on the affinity of C-terminal residues for HLA-B*57:01. Values are represented as geometric mean with 95% CI of the fold difference between vehicle/acyclovir treatment. The experiment was run 6 times with each run performed in triplicates. Analyzed for statistical significance by column statistics; p < 0.05 was considered significant (*p < 0.05; **p < 0.01; ***p < 0.001). The most pronounced affinity increases for HLA-B*57:01 in the presence of 2 mg/mL of acyclovir were found for peptides with a cysteine, isoleucine and valine at the C-terminus.

When the PSCPL was screened, it was found that the greatest increases in HLA-B*57:01 affinity, in the context of acyclovir treatment, were associated with the presence of C, I and V at the C-terminus (Fig 4). For each of these residues, a 2-fold increase in B*57:01 affinity was observed in the presence of the acyclovir compared to a 10-fold increase in affinity for I and V peptides previously observed in the presence of abacavir [4]. The C-terminal peptide library with the highest affinity for HLA-B*57:01 in the presence of acyclovir was observed for isoleucine (478 nM in the presence of acyclovir vs. 1470 nM in the absence) followed by valine and cysteine (Table 1). In conclusion, the data in this section have shown that the PSCPL could detect an acyclovir-induced effect on the specificity of HLA-B*57:01 binding based on modified affinity for peptides with C, I and V terminal residues.

**Table 1:** Peptide affinity (nM) for HLA-B*57:01 in the presence of 2 mg/mL acyclovir.

Peptide	IC_50_ (vehicle)	IC_50_ (+acyclovir)
XXXXXXXXA	6150	4970
**XXXXXXXXC**	7830	**4330**
XXXXXXXXD	23300	12900
XXXXXXXXE	10100	7600
XXXXXXXXF	127	163
XXXXXXXXG	22200	15800
XXXXXXXXH	13900	29200
**XXXXXXXXI**	1470	**478**
XXXXXXXXK	25800	9810
XXXXXXXXL	1280	1160
XXXXXXXXM	895	697
XXXXXXXXN	26000	26900
XXXXXXXXP	62100	68100
XXXXXXXXQ	39400	37200
XXXXXXXXR	14700	16200
XXXXXXXXS	11900	22000
XXXXXXXXT	22000	31400
**XXXXXXXXV**	5740	**3360**
XXXXXXXXW	109	134
XXXXXXXXY	19000	13400

Bold indicates peptides that showed a significant increase in affinity for HLA-B*57:01 in the presence of acyclovir (as shown in Fig 4) and also had an IC_50_ ≤ 5,000 nM. Values are derived from the medians of six independent measurements run in triplicates.

### Follow up on PSCPL with individual C-terminal I and C peptides

**Fig 5:**
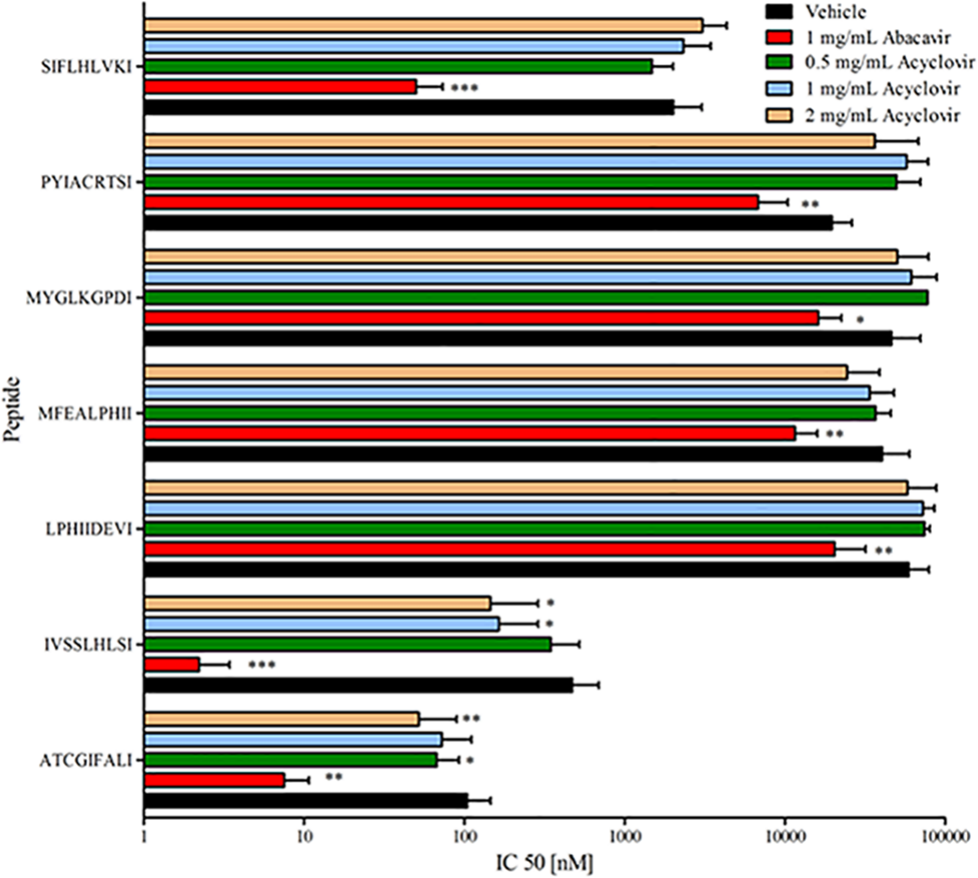
Effects of abacavir and acyclovir on HLA-B*57:01 binding specificity. Specific peptides with a terminal isoleucine that showed an increased affinity for HLA-B*57:01 in the presence of abacavir were tested. Values are represented as geometric mean with 95% CI of two independent runs in triplicates, analyzed for statistical significance by Mann-Whitney U test comparing log IC_50_ values vs. vehicle; p < 0.05 was considered significant (*p < 0.05; **p < 0.01; ***p < 0.001).

To be able to induce an immune response that can lead to an ADR, a peptide has to bind MHC molecules with a high enough affinity to ensure efficient presentation to T cells. For this reason we were particularly interested in investigating ligands with C-terminal residues associated with affinities, as measured with the PSCPL, of 5,000 nM or better. In addition to peptides with C-terminal V that we had already tested in our initial screening (Fig 3) we also observed a significant increase in B*57:01 binding affinity for I and C terminal residues in the presence of acyclovir (Table 1). Accordingly, we selected 7 peptides with either C or I at their C-terminus and tested them for binding to B*57:01 with and without acyclovir. The only requirement for these peptides was that they had a C or I at P9 but were otherwise random in sequence. Binding to HLA-B*57:01 in the presence of abacavir was used as a positive control. As shown in Fig 5, of the 7 peptides ending in I, only two showed a dose dependent increase in affinity for HLA-B*57:01. By contrast, all seven peptides showed increased affinities in the presence of abacavir. None of the peptides ending in C showed any change in HLA-B*57:01 affinity in the presence of acyclovir (S4 Fig). Thus, these data show that the acyclovir-induced effect on HLA-B*57:01 specificity detected with the PSCPL could also be detected in the context of individual peptides with isoleucine but not for those with cysteine C-termini.

### Direct drug binding assays

**Fig 6:**
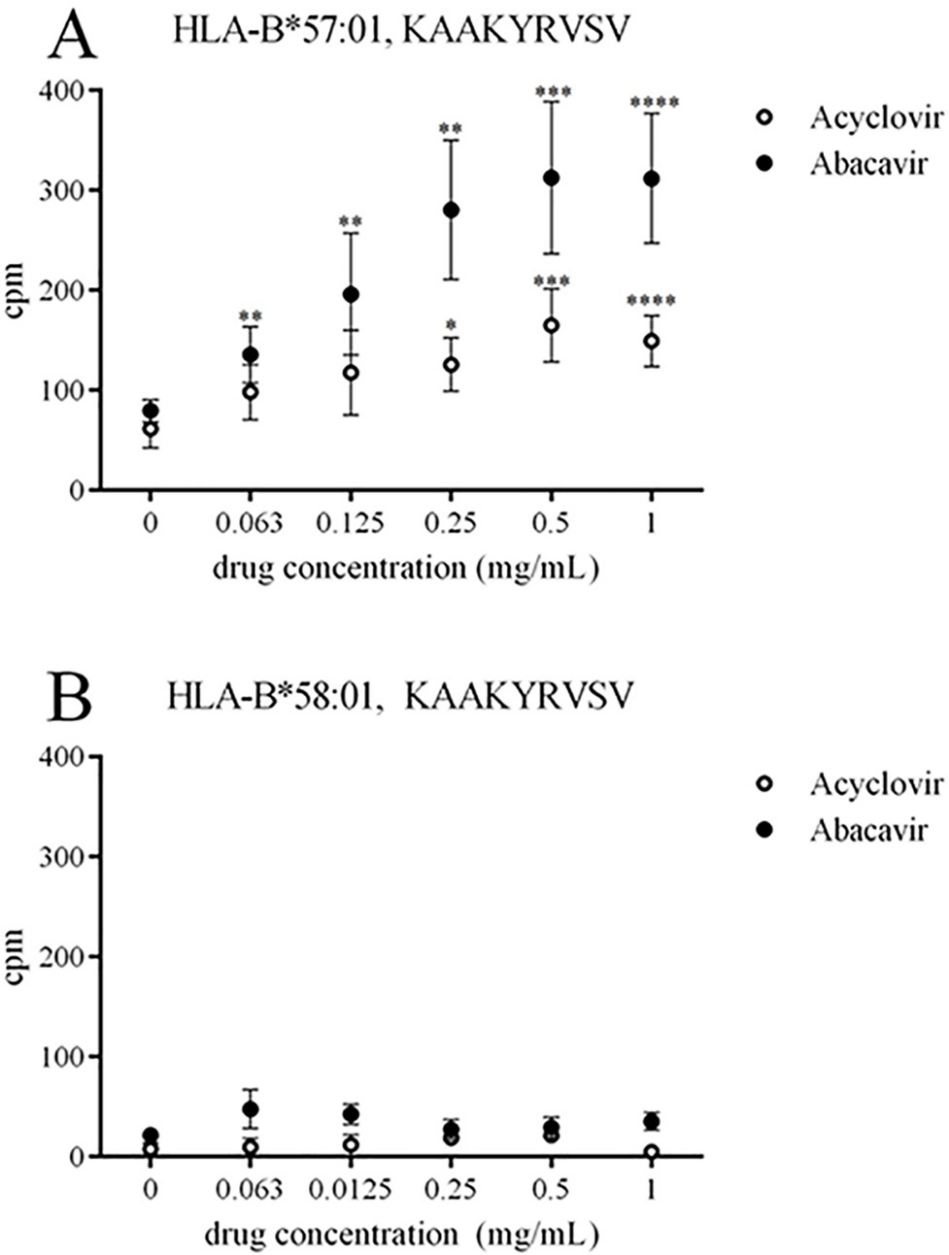
Acyclovir and abacavir alter the binding specificity of HLA-B*57:01. The peptide KAAKYRVSV was radiolabeled and tested for binding to HLA-B*57:01 in increasing doses of acyclovir and abacavir. Values are represented as geometric mean with 95% CI of four experimental runs in triplicates. Analysed by statistical significance by one-sided Mann Whitney test. *p<0.05, **p<0.01, ***p<0.001, ****p<0.0001.

All binding data reported so far relied on detecting replacement of a labeled competitor peptide. To measure direct binding of peptide to MHC we synthesized a variant of peptide KAAKIRVSV in which the isoleucine residue in position 5 was exchanged with a tyrosine residue to enable radiolabeling. The variant peptide with the tyrosine substitution bound with six-fold lower affinity in the presence of 1 mg/mL acyclovir (IC-50: 10040 ± 2336 for KAAKYRVSV vs. 1560 ± 865 for KAAKIRVSV; results represented ad median ± SD). The direct peptide binding assay demonstrated that peptide KAAKYRVSV binds with increasing efficiency to HLA-B*57:01 in a dose-dependent manner (Fig 6A). As expected, the presence of abacavir was associated with an even greater effect, resulting in an up to 5-fold increase in bound peptides, while acyclovir increased the level of bound peptides 2-fold (Fig 6A). In contrast, as a control, when binding to the structurally similar HLA-B*58:01 molecule was examined, KAAKYRVSV was found to be unaffected by the presence of either acyclovir or abacavir (Fig 6B).

### Treatment of HLA-B*57:01 Cell Line with Acyclovir Results in an Increased Number of Peptides with C-terminal Isoleucine

Our binding studies with the combinatorial libraries predicted that HLA-B*57:01 in the presence of acyclovir would favor presentation of peptides with cysteine, isoleucine or valine at their C-terminus, and this was validated for individual peptides for isoleucine and valine. To test this prediction, we eluted peptides from an HLA-B*57:01 single allele transfected cell line in the presence or absence of acyclovir. The identity of eluted peptides was determined by mass spectrometry and comparing the gathered spectra against the Swiss-Prot human database as described in the Methods. Of note, the MS detection by itself cannot distinguish isoleucine from leucine residues, so distinguishing peptides with these sequences relies on the database comparison to human proteins. We identified 169 and 95 unique sequences from drug treated and untreated samples, respectively, and an additional 91 sequences that were present in both samples (S1 Table). Next, we analyzed the eluted peptide sequences for specific characteristics. No peptides with a C-terminal cysteine or valine were identified in either of the samples (S1 Table). In contrast, we did identify a higher number of peptides with a C-terminal isoleucine in the presence of acyclovir (twelve) compared to the untreated samples (four), with three of the peptides being present in both samples (S1 & S2 Tables). However, this difference was not significant (p = 0.073, Fisher’s exact test).

For the nine peptides with a C-terminal isoleucine that were initially exclusively identified in the drug treated sample in the combined MS and database search, we performed targeted queries to determine if they could have been present in the untreated sample. These searches revealed that one peptide with the assigned sequence RARQLNYTI was exclusively found in the drug treated sample in that it was not selected for MS2 in the untreated sample, nor was it seen in the MS1 scan when searched for by accurate mass. When this peptide was ordered and tested for its capacity to bind treated and untreated HLA-B*57:01 it was found that binding was indeed improved by about two-fold in the presence of acyclovir (S5 Fig; IC-50: 13.7 ± 1.3 for vehicle vs. 7.1 ± 1.0 for 0.5 mg/mL acyclovir; Mean ± SEM, **p < 0.01). These studies illustrate that the elution of peptides from HLA-B*57:01 transfected cell lines in the presence of acyclovir is consistent with the *in vitro* PSCPL and individual peptide data.

### Acyclovir is unable to Induce *in vitro* HLA-B*57:01 Restricted T-cell Responses

**Fig 7:**
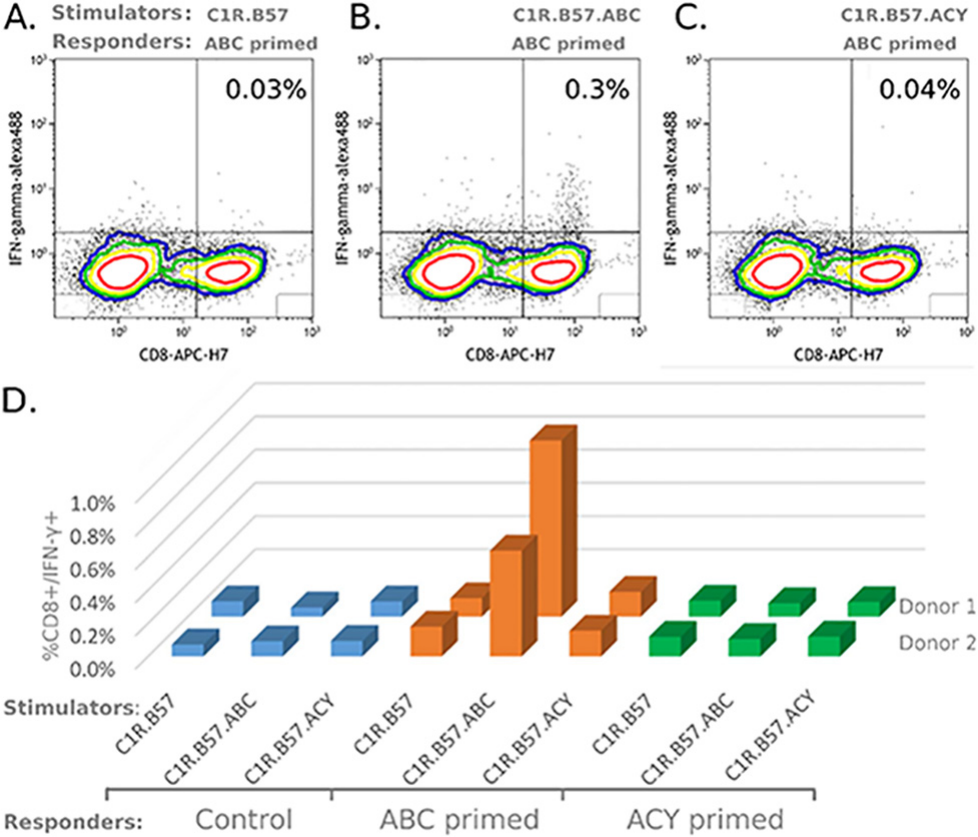
PBMC from a healthy HLA-B*57:01 positive donor (Donor 1) where primed with abacavir at day 0, cultured for 14 days and then restimulated 1:10 with (A) HLA-B*57:01 single antigen line (C1R.B57), (B) with O/N abacavir treated C1R.B57 (C1R.B57.ABC) or (C) with O/N acyclovir treated C1R.B57 (C1R.B57.ACY). Antigen activated cells were detected by ICS for IFN-γ production and CD8+/ IFN-γ T-cells quantitated using flow cytometry. (D) PBMC from two healthy HLA-B*57:01 positive donors were either primed with abacavir (ABC primed), primed with acyclovir (ACY primed) or had no treatment (Control. PBMC were cultured for 14 days and then stimulated 1:10 with treated and untreated single antigen line, C1R.B57, as indicated.

We and others have previously reproduced abacavir specific CD8^+^ T cell responses in short-term lines from 100% of HLA-B*57:01^+^ abacavir unexposed healthy blood donors [20]. To investigate whether acyclovir has the ability to induce T-cell responses in a HLA restricted manner *in vitro*, we compared the effect of acyclovir, abacavir or no drug treatment, on the generation of drug specific short term lines, using PBMC from two healthy HLA-B*57:01 positive, HIV-negative and abacavir unexposed donors. Drug specific responses were assessed following re-stimulation of 14 day cultures with a drug treated or untreated HLA-B*57:01 stably transfected antigen presenting cell line, C1R.B57, and the frequency of CD8 positive / IFN-γ positive T-cells was quantitated using flow cytometry. As expected, we detected an expanded population of CD8 positive / IFN-γ positive T-cells in abacavir primed lines restimulated by abacavir-treated C1R.B57 (Fig 7B), but not following re-stimulation with untreated (Fig 7A) or acyclovir-treated C1R.B57 (Fig 7C), in two of two donors. We could not detect any acyclovir specific response in PBMC cultures primed with acyclovir (Fig 7D), cultured for 14 days and re-stimulated with acyclovir treated C1R.B57 in two of two donors. Similarly, we could not detect any stimulation of acyclovir primed cultures using untreated or abacavir treated C1R.B57 in two of two donors (Fig 7D). This suggests that under *in-vitro* conditions, acyclovir does not have the capacity to stimulate T-cells.

## Discussion

There is an unmet need for sensitive and specific pre-clinical screening methodologies to efficiently identify drug candidates that might cause IM-ADRs in early stages of drug design and development. Recently, our group and others have identified a new mechanism of how the small molecule abacavir interacts exclusively with HLA B*57:01 molecules, leading to an alteration in the HLA-B*57:01 peptide binding repertoire, and a drug-associated immune mediated T-cell hypersensitivity reaction [4–6]. The assays utilized in these studies have the potential to act as screening tools to assess drugs for similar effects. To assess if there is indeed a correlation between clinical safety and the readouts of these assays, we here examined drugs with established clinical safety profiles that are structurally similar to abacavir. Of the panel of drugs tested, we found that acyclovir had the strongest effect and did alter the binding specificity and ligand repertoire of HLA-B*57:01 in a qualitatively similar fashion as abacavir, although quantitatively to a much lower degree. Similar to abacavir, acyclovir is a guanosine analogue currently used as an antiviral agent. Acyclovir, however, has a robust safety record with almost 30 years of post-marketing experience, unlike abacavir has not been associated with any treatment limiting IM-ADRs except for isolated case reports [21–24]. Thus, the changes induced by acyclovir to the binding specificity and ligand repertoire of HLA-B*57:01 are not in themselves sufficient to trigger IM-ADRs.

Quantitatively, the presence of acyclovir led to 2–3 fold increases in binding affinity as measured by IC50 and to the presentation of a single peptide that was clearly absent in untreated cells. In contrast, abacavir led to 1,000 fold increases in IC50 values for individual peptides and large numbers of peptides presented exclusively in the presence of the drug (such as 15 peptides with a C-terminal valine). Given the safety profile of acyclovir, we suggest that effects of the magnitude observed here are below the threshold of what can be considered safe in terms of induction of an altered peptide repertoire based IM-ADR. Thus, this study establishes a first estimate of a threshold of what can be considered a safe in the assays utilized here.

Based on our binding studies, one could have expected that peptides with a C-terminal Cysteine would preferentially be presented and eluted in the presence of acyclovir. However, peptides with c-terminal cysteine residues are poor substrates for both proteasomal cleavage and TAP binding, and are found at low frequencies in peptide elution studies (e.g. 2/935 = 0.03%) [25]. Given the small effect of acyclovir, we were not surprised that it did not overcome this barrier to presentation.

In addition to the binding and elution assays, we also performed cell culture studies in which we examined if we can induce drug responsive T-cells in HLA-B*57:01^+^ PBMCs cultures from abacavir naive healthy individuals. These studies suggest that abacavir, but not acyclovir, can induce drug response T cells after a two-week culture. Such cell culture assays have the advantage of requiring less specialized instrumentation and reagents compared to the radio-immuno binding assay or the mass spectrometry coupled elution experiments.

One limitation of our study was that, in the absence of identifying a drug that showed strong abacavir-like effects, we cannot say for certain that the structure based drug selection strategy applied was successful. Future studies will examine if different selection strategies or screening compounds at a large scale might have identified drugs with similar or larger effects than acyclovir. Furthermore, most drugs that were predicted to have structural similarity to abacavir showed no effect in our binding assays. Given that we are unable to measure direct binding of the drugs to HLA, we cannot know if these results are due to the drugs not binding to HLA-B57:01. We do consider such lack of binding the most likely hypothesis given the rather constrained binding site of abacavir in the crystal structure, which could mean that even similar drugs are too different from abacavir to fit into the specific site. More importantly, we consider such lack of binding to be a more likely explanation compared to the alternative hypothesis that the drugs do bind to HLA-B57:01 but do not alter the binding specificity of the molecule, which seems structurally unlikely given the fact C-terminal peptide residue side chains do overlap with the site occupied by abacavir.

In conclusion, we have here compared different *in vitro* readouts that could be potential indicators of HLA-associated IM-ADRs. Notably, the clinically safe drug acyclovir which is structurally very similar to abacavir showed qualitative evidence of interaction with HLA-B*57:01 but little or no quantitative or functional effect. We suggest that these *in vitro* approaches show great promise as a pre-clinical screening strategy if they can be developed further to establish safety thresholds that can be validated with high sensitivity and specificity to predict altered self-repertoire mediated IM-ADRs.

## Supporting Information

S1 Fig (left) The structure of the co-crystallized abacavir-peptide-MHC molecule (PDB ID: 3UPR): abacavir shown in green and peptide (HSITYLLPV) shown in red; (right) structure of the acyclovir-peptide-MHC molecule predicted by molecular docking studies (GOLD 5.2) acyclovir shown in blue and peptide (HSITYLLPV) shown in red. Figure prepared by using PyMOL (The PyMOL Molecular Graphics System, Version 1.5.0.4 Schrödinger, LLC.).
(TIF)

S2 Fig Illustration of the binding patterns between the MHC molecule and acyclovir predicted by molecular docking studies. Hydrogen bond interactions between the Ile 124 (MHC molecule) main chains and acyclovir is represented by green dashed arrow. Hydrogen bond interactions between side-chains of Ser 116, Asp 114 (MHC molecule) and acyclovir is represented by blue dashed arrow. Pi interactions between the Trp147, Tyr74 of the MHC molecule and acyclvir is shown in orange line. Figure prepared by using Discovery Studio software (version 3.1; Accelrys Inc., USA).
(TIF)

S3 Fig Effect of seven drugs on binding specificity of HLA-B*57:01. Three different peptides were tested for binding to HLA-B*57:01 in competitive binding assays as described previously [26,27]. Values are represented as geometric mean with 95% CI of one experimental run in quadruplicates, analyzed for statistical significance by paired one-tailed parametric t-test comparing log IC_50_ values vs. vehicle; *p < 0.05 was significant only in the presence of acyclovir.
(TIF)

S4 Fig Effects of abacavir and acyclovir on HLA-B*57:01 binding specificity. Specific peptides with a terminal cysteine were tested for their binding affinity in the presence of abacavir or acyclovir. Values are represented as geometric mean with 95% CI of two independent runs in triplicates, analyzed for statistical significance by Mann-Whitney U test comparing log IC_50_ values vs. vehicle; p < 0.05 was considered significant (*p < 0.05; **p < 0.01; ***p < 0.001).
(TIF)

S5 Fig Effects of acyclovir on HLA-B*57:01 binding specificity for RARQLNYTI. Values are represented as Mean ± SEM and analyzed for statistical significance by Mann-Whitney U test comparing IC_50_ values vs. vehicle; p < 0.05 was considered significant (**p < 0.01).
(TIF)

S1 Table List of peptides eluted in the presence and absence of acyclovir. (DOCX)

S2 Table Number of peptides with C terminal isoleucine in ACI treated vs. untreated cells. (DOCX)
