# Supplementary figures and images for: Acyclovir Has Low but Detectable Influence on HLA-B*57:01 Specificity without Inducing Hypersensitivity
Source: PLoS One. 2015 May 29;10(5):e0124878. doi: 10.1371/journal.pone.0124878 (PMC4449000; doi:10.1371/journal.pone.0124878)

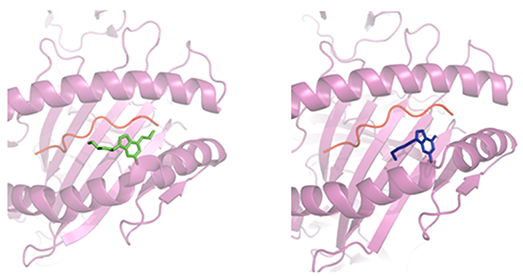

Supplement: S1 Fig — Figure prepared by using PyMOL (The PyMOL Molecular Graphics System, Version 1.5.0.4 Schrödinger, LLC.). (TIF) [file pone.0124878.s001.tif]

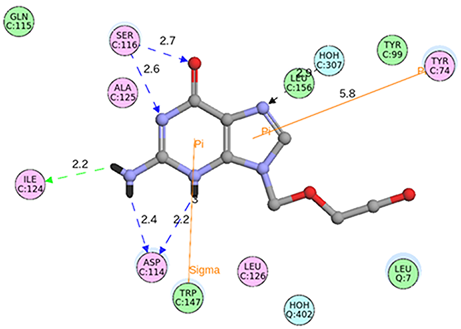

Supplement: S2 Fig — Hydrogen bond interactions between the Ile 124 (MHC molecule) main chains and acyclovir is represented by green dashed arrow. Hydrogen bond interactions between side-chains of Ser 116, Asp 114 (MHC molecule) and acyclovir is represented by blue dashed arrow. Pi interactions between the Trp147, Tyr74 of the MHC molecule and acyclvir is shown in orange line. Figure prepared by using Discovery Studio software (version 3.1; Accelrys Inc., USA). (TIF) [file pone.0124878.s002.tif]

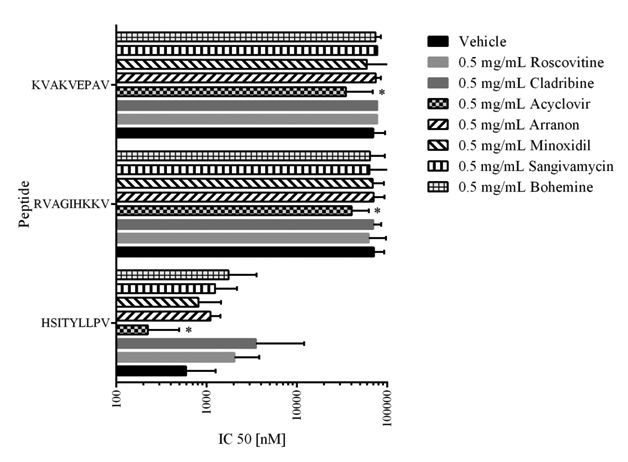

Supplement: S3 Fig — Three different peptides were tested for binding to HLA-B*57:01 in competitive binding assays as described previously [26,27]. Values are represented as geometric mean with 95% CI of one experimental run in quadruplicates, analyzed for statistical significance by paired one-tailed parametric t-test comparing log IC50 values vs. vehicle; *p < 0.05 was significant only in the presence of acyclovir. (TIF) [file pone.0124878.s003.tif]

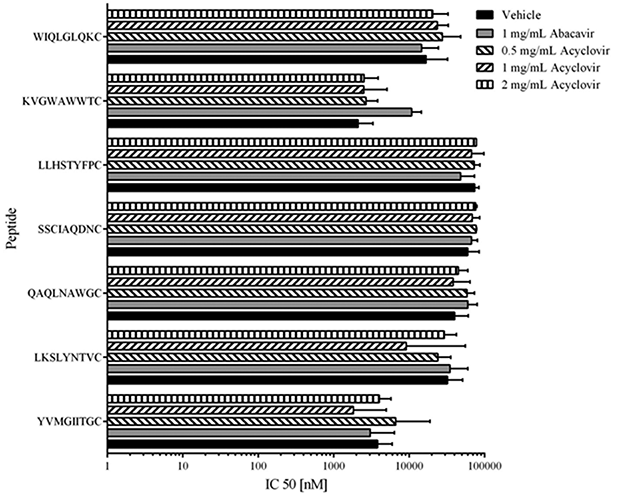

Supplement: S4 Fig — Specific peptides with a terminal cysteine were tested for their binding affinity in the presence of abacavir or acyclovir. Values are represented as geometric mean with 95% CI of two independent runs in triplicates, analyzed for statistical significance by Mann-Whitney U test comparing log IC50 values vs. vehicle; p < 0.05 was considered significant (*p < 0.05; **p < 0.01; ***p < 0.001). (TIF) [file pone.0124878.s004.tif]

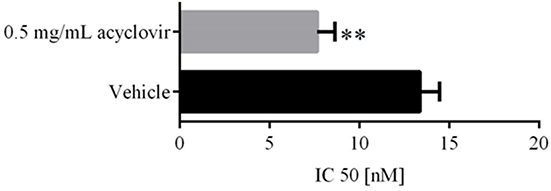

Supplement: S5 Fig — Values are represented as Mean ± SEM and analyzed for statistical significance by Mann-Whitney U test comparing IC50 values vs. vehicle; p < 0.05 was considered significant (**p < 0.01). (TIF) [file pone.0124878.s005.tif]
