# Supplementary material for: Acyclovir Has Low but Detectable Influence on HLA-B*57:01 Specificity without Inducing Hypersensitivity
Source: PLoS One. 2015 May 29;10(5):e0124878. doi: 10.1371/journal.pone.0124878 (PMC4449000; doi:10.1371/journal.pone.0124878)
Supplement: S1 Table — (DOCX) [file pone.0124878.s006.docx]

Table S1: List of peptides eluted in the presence and absence of acyclovir

| **Sample** | **Peptide Sequence** | **C-term** | **Length** | **Accession** | **Source Protein** | **Start** | **Stop** |
| --- | --- | --- | --- | --- | --- | --- | --- |
| both | KSRGVLHQF | F | 9 | P27144 | Adenylate kinase isoenzyme 4, mitochondrial | 186 | 194 |
| both | KSRPIIKRF | F | 9 | Q76N89 | E3 ubiquitin-protein ligase HECW1 | 289 | 297 |
| both | KTRTNVPTF | F | 9 | Q96BU1 | S100P-binding protein | 305 | 313 |
| both | RSLRLQKQF | F | 9 | Q12788 | Transducin beta-like protein 3 | 322 | 330 |
| both | RTQIRHMKF | F | 9 | O95503 | Chromobox protein homolog 6 | 221 | 229 |
| both | RTVEKPPKF | F | 9 | Q01082 | Spectrin beta chain, brain 1 | 348 | 356 |
| both | TTLEHQKTF | F | 9 | Q13347 | Eukaryotic translation initiation factor 3 subunit I | 218 | 226 |
| both | YTYRGPKAF | F | 9 | Q12874 | Splicing factor 3A subunit 3 | 414 | 422 |
| both | KSRDYEREI | I | 9 | Q9NRZ9 | Lymphoid-specific helicase | 776 | 784 |
| both | KTFTTQETI | I | 9 | P06744 | Glucose-6-phosphate isomerase | 211 | 219 |
| both | KTRQIISTI | I | 9 | P37268 | Squalene synthase | 358 | 366 |
| both | HTWNGIRHL | L | 9 | Q99643 | Succinate dehydrogenase cytochrome b560 subunit, mitochondrial | 127 | 135 |
| both | KAMELIREL | L | 9 | Q14691 | DNA replication complex GINS protein PSF1 | 5 | 13 |
| both | ETKKKVFLW | W | 9 | Q86V20 | Protein FAM35A | 481 | 489 |
| both | ISKEEAMRW | W | 9 | P62913 | 60S ribosomal protein L11 | 157 | 165 |
| both | ISKRLGKRW | W | 9 | Q06945 | Transcription factor SOX-4 | 89 | 97 |
| both | ITAGAHRLW | W | 9 | O00767 | Acyl-CoA desaturase | 115 | 123 |
| both | ITKKQVSVW | W | 9 | P13196 | 5-aminolevulinate synthase, nonspecific, mitochondrial | 240 | 248 |
| both | ITTKAISRW | W | 9 | P49368 | T-complex protein 1 subunit gamma | 160 | 168 |
| both | KAGQVVTIW | W | 9 | P02545 | Prelamin-A/C | 490 | 498 |
| both | KAKTNVKLW | W | 9 | P10644 | cAMP-dependent protein kinase type I-alpha regulatory subunit | 216 | 224 |
| both | KGKPVRIMW | W | 9 | Q9H361 | Polyadenylate-binding protein 3 | 78 | 86 |
| both | KTKEVIQEW | W | 9 | Q9Y490 | Talin-1 | 343 | 351 |
| both | KTKEVLQEW | W | 9 | Q9Y4G6 | Talin-2 | 346 | 354 |
| both | RQSTILKRW | W | 9 | Q96CS7 | Pleckstrin homology domain-containing family B member 2 | 11 | 19 |
| both | RTVIIEQSW | W | 9 | P10809 | 60 kDa heat shock protein, mitochondrial | 60 | 68 |
| both | SADKTVKLW | W | 9 | Q9Y263 | Phospholipase A-2-activating protein | 170 | 178 |
| both | SVTEIQEKW | W | 9 | Q9NR30 | Nucleolar RNA helicase 2 | 689 | 697 |
| both | TAKISDFSW | W | 9 | Q16576 | Histone-binding protein RBBP7 | 373 | 381 |
| both | TSKITSTAW | W | 9 | Q9UKA1 | F-box/LRR-repeat protein 5 | 423 | 431 |
| both | VTKKTYEIW | W | 9 | Q14839 | Chromodomain-helicase-DNA-binding protein 4 | 1721 | 1729 |
| both | VTNPHSSQW | W | 9 | P24522 | Growth arrest and DNA damage-inducible protein GADD45 alpha | 127 | 135 |
| both | YTDNLVRVW | W | 9 | P63244 | Guanine nucleotide-binding protein subunit beta-2-like 1 | 302 | 310 |
| both | ISKALVAYY | Y | 9 | P62249 | 40S ribosomal protein S16 | 88 | 96 |
| both | KSFVKVYNY | Y | 9 | P61353 | 60S ribosomal protein L27 | 69 | 77 |
| both | KTKEIEQVY | Y | 9 | Q8NEY8 | Periphilin-1 | 297 | 305 |
| both | KTRIIDVVY | Y | 9 | P62241 | 40S ribosomal protein S8 | 75 | 83 |
| both | MTNPHNHLY | Y | 9 | Q15006 | Tetratricopeptide repeat protein 35 | 183 | 191 |
| both | RTIVIRRDY | Y | 9 | P62280 | 40S ribosomal protein S11 | 84 | 92 |
| both | RTVRIWRQY | Y | 9 | O76071 | Probable cytosolic iron-sulfur protein assembly protein CIAO1 | 216 | 224 |
| **Sample** | **Peptide Sequence** | **C-term** | **Length** | **Accession** | **Source Protein** | **Start** | **Stop** |
| both | RVRDVVTKY | Y | 9 | Q12931 | Heat shock protein 75 kDa, mitochondrial | 269 | 277 |
| both | KSRGYVKEQF | F | 10 | P46783 | 40S ribosomal protein S10 | 53 | 62 |
| both | KSSQVQRRFF | F | 10 | P13010 | X-ray repair cross-complementing protein 5 | 347 | 356 |
| both | KTKDGVREVF | F | 10 | P61586 | Transforming protein RhoA | 162 | 171 |
| both | KTKEGVREVF | F | 10 | P62745 | Rho-related GTP-binding protein RhoB | 162 | 171 |
| both | KTMKMRGQAF | F | 10 | P08579 | U2 small nuclear ribonucleoprotein B'' | 44 | 53 |
| both | VTKTVSNDSF | F | 10 | P55209 | Nucleosome assembly protein 1-like 1 | 288 | 297 |
| both | AAADSAVRLW | W | 10 | Q6IA86 | Elongator complex protein 2 | 133 | 142 |
| both | ATRKHGTDLW | W | 10 | Q9NP61 | ADP-ribosylation factor GTPase-activating protein 3 | 124 | 133 |
| both | HGKPVTQVTW | W | 10 | Q14137 | Ribosome biogenesis protein BOP1 | 533 | 542 |
| both | HSAKVHSVAW | W | 10 | Q96J01 | THO complex subunit 3 | 54 | 63 |
| both | HSITSDNHKW | W | 10 | Q8NEM7 | Protein FAM48A | 178 | 187 |
| both | ITAENVAKKW | W | 10 | Q9BWD1 | Acetyl-CoA acetyltransferase, cytosolic | 162 | 171 |
| both | ITKADAAEFW | W | 10 | P22681 | E3 ubiquitin-protein ligase CBL | 181 | 190 |
| both | ITKEIEANEW | W | 10 | O75410 | Transforming acidic coiled-coil-containing protein 1 | 615 | 624 |
| both | ITSQDVLHSW | W | 10 | P00403 | Cytochrome c oxidase subunit 2 | 154 | 163 |
| both | KAFDEKKQKW | W | 10 | Q53T94 | TATA box-binding protein-associated factor RNA polymerase I subunit B | 409 | 418 |
| both | KGFSEEHNTW | W | 10 | P45973 | Chromobox protein homolog 5 | 42 | 51 |
| both | KSAEKEISLW | W | 10 | P22392 | Nucleoside diphosphate kinase B | 124 | 133 |
| both | KSFEKAKESW | W | 10 | Q13451 | Peptidyl-prolyl cis-trans isomerase FKBP5 | 248 | 257 |
| both | KSGPVVSLGW | W | 10 | Q9H269 | Vacuolar protein sorting-associated protein 16 homolog | 82 | 91 |
| both | KSSIAGSSTW | W | 10 | Q99808 | Equilibrative nucleoside transporter 1 | 315 | 324 |
| both | LTRKMRRDGW | W | 10 | P17844 | Probable ATP-dependent RNA helicase DDX5 | 357 | 366 |
| both | RSASPSSQGW | W | 10 | Q5VV67 | Peroxisome proliferator-activated receptor gamma coactivator-related protein 1 | 1410 | 1419 |
| both | RSFKETTNRW | W | 10 | Q92613 | Protein Jade-3 | 714 | 723 |
| both | RSVGRISKQW | W | 10 | Q9NRY6 | Phospholipid scramblase 3 | 231 | 240 |
| both | RSYSESEKQW | W | 10 | Q96HQ2 | CDKN2AIP N-terminal-like protein | 28 | 37 |
| both | RTILVDNNTW | W | 10 | P46977 | Dolichyl-diphosphooligosaccharide--protein glycosyltransferase subunit STT3A | 538 | 547 |
| both | RTTLVDNNTW | W | 10 | Q8TCJ2 | Dolichyl-diphosphooligosaccharide--protein glycosyltransferase subunit STT3B | 617 | 626 |
| both | TAAQITQRKW | W | 10 | P30480 | HLA class I histocompatibility antigen, B-42 alpha chain | 162 | 171 |
| both | TSSKPDPSQW | W | 10 | O75410 | Transforming acidic coiled-coil-containing protein 1 | 380 | 389 |
| both | VTNKSQIRTW | W | 10 | P27694 | Replication protein A 70 kDa DNA-binding subunit | 203 | 212 |
| both | KTVAGGAWTY | Y | 10 | P61513 | 60S ribosomal protein L37a | 62 | 71 |
| both | ITSSIHSKETF | F | 11 | Q07864 | DNA polymerase epsilon catalytic subunit A | 1890 | 1900 |
| both | KSRGSNLRVHF | F | 11 | P18621 | 60S ribosomal protein L17 | 16 | 26 |
| both | HSGPRGTHDLW | W | 11 | Q04446 | 1,4-alpha-glucan-branching enzyme | 312 | 322 |
| both | HTNQDHVHAVW | W | 11 | Q9UNY4 | Transcription termination factor 2 | 526 | 536 |
| both | KSKAMTGVEQW | W | 11 | P49368 | T-complex protein 1 subunit gamma | 425 | 435 |
| both | KSKLDAEVSKW | W | 11 | P35221 | Catenin alpha-1 | 695 | 705 |
| both | KTVDGPSGKLW | W | 11 | P04406 | Glyceraldehyde-3-phosphate dehydrogenase | 186 | 196 |
| both | KVIHEQVNHRW | W | 11 | P11388 | DNA topoisomerase 2-alpha | 287 | 297 |
| **Sample** | **Peptide Sequence** | **C-term** | **Length** | **Accession** | **Source Protein** | **Start** | **Stop** |
| both | RAFQAHKEENW | W | 11 | Q5JVF3 | PCI domain-containing protein 2 | 93 | 103 |
| both | RVFEDESGKHW | W | 11 | Q8TEA8 | D-tyrosyl-tRNA(Tyr) deacylase 1 | 53 | 63 |
| both | SASPDATIRIW | W | 11 | Q9UMS4 | Pre-mRNA-processing factor 19 | 281 | 291 |
| both | SSATDAAIRVW | W | 11 | Q12788 | Transducin beta-like protein 3 | 170 | 180 |
| both | VAKKTKDVTVW | W | 11 | Q96DR4 | StAR-related lipid transfer protein 4 | 31 | 41 |
| both | VGLPAAGKTTW | W | 11 | Q9BUJ2 | Heterogeneous nuclear ribonucleoprotein U-like protein 1 | 427 | 437 |
| both | RTKKVGIVGKY | Y | 11 | P61513 | 60S ribosomal protein L37a | 4 | 14 |
| both | RSKDDPGKGSYW | W | 12 | Q9UPW0 | Forkhead box protein J3 | 141 | 152 |
| both | VSDSGAHVLNSW | W | 12 | Q9NVM9 | Protein asunder homolog | 80 | 91 |
| both | KVKDGPGGKEATW | W | 13 | P22307 | Non-specific lipid-transfer protein | 462 | 474 |
| treated | RTRIGYSF | F | 8 | Q9NYH9 | U3 small nucleolar RNA-associated protein 6 homolog | 72 | 79 |
| treated | VTTIARNW | W | 8 | Q15436 | Protein transport protein Sec23A | 496 | 503 |
| treated | GTHKVTVLF | F | 9 | P21333 | Filamin-A | 352 | 360 |
| treated | ISKKINTKF | F | 9 | O00232 | 26S proteasome non-ATPase regulatory subunit 12 | 205 | 213 |
| treated | ITKRKEVIF | F | 9 | Q8NCY6 | Coiled-coil domain-containing protein KIAA1826 | 24 | 32 |
| treated | KSSEVFTTF | F | 9 | P40222 | Alpha-taxilin | 403 | 411 |
| treated | KSVTLGYLF | F | 9 | P55265 | Double-stranded RNA-specific adenosine deaminase | 1063 | 1071 |
| treated | KTAPFDSRF | F | 9 | P14854 | Cytochrome c oxidase subunit 6B1 | 13 | 21 |
| treated | KTRLVREGF | F | 9 | Q9Y2P5 | Bile acyl-CoA synthetase | 645 | 653 |
| treated | KTSGIINKF | F | 9 | O95243 | Methyl-CpG-binding domain protein 4 | 328 | 336 |
| treated | LSSPVTKSF | F | 9 | P01834 | Ig kappa chain C region | 93 | 101 |
| treated | MTKRDVPGF | F | 9 | Q9BXR0 | Queuine tRNA-ribosyltransferase | 217 | 225 |
| treated | RAKAIIVEF | F | 9 | O60841 | Eukaryotic translation initiation factor 5B | 790 | 798 |
| treated | RSFHEVREF | F | 9 | Q92963 | GTP-binding protein Rit1 | 106 | 114 |
| treated | RTATFQQRF | F | 9 | Q9BY77 | Polymerase delta-interacting protein 3 | 46 | 54 |
| treated | RTKQVLHTF | F | 9 | Q9BTV5 | Fibronectin type III and SPRY domain-containing protein 1 | 439 | 447 |
| treated | RTSIVEKRF | F | 9 | Q9BXR0 | Queuine tRNA-ribosyltransferase | 361 | 369 |
| treated | RTTNLIRHF | F | 9 | Q14592 | Zinc finger protein 460 | 459 | 467 |
| treated | TSKPVQMMF | F | 9 | P48595 | Serpin B10 | 213 | 221 |
| treated | VTKKIKVEF | F | 9 | Q8NEN9 | PDZ domain-containing protein 8 | 119 | 127 |
| treated | KARELDPRI | I | 9 | Q9ULW0 | Targeting protein for Xklp2 | 403 | 411 |
| treated | KEKLIFSEI | I | 9 | Q8WTR4 | Glycerophosphodiester phosphodiesterase domain-containing protein 5 | 550 | 558 |
| treated | KSKNILFVI | I | 9 | Q9H009 | Nascent polypeptide-associated complex subunit alpha-2 | 98 | 106 |
| treated | KSVLVKQTI | I | 9 | Q9UHX1 | Poly(U)-binding-splicing factor PUF60 | 80 | 88 |
| treated | KTRDYLIKI | I | 9 | Q99666 | RANBP2-like and GRIP domain-containing protein 5/6 | 713 | 721 |
| treated | RARQLNYTI | I | 9 | O60762 | Dolichol-phosphate mannosyltransferase | 216 | 224 |
| treated | KTFRIKRFL | L | 9 | P62891 | 60S ribosomal protein L39 | 5 | 13 |
| treated | KTFVGRAKL | L | 9 | Q9Y4K1 | Absent in melanoma 1 protein | 301 | 309 |
| treated | KTHAVLVAL | L | 9 | P25786 | Proteasome subunit alpha type-1 | 41 | 49 |
| treated | KTKEAVLLL | L | 9 | P36578 | 60S ribosomal protein L4 | 163 | 171 |
| treated | KTLERSYLL | L | 9 | P23921 | Ribonucleoside-diphosphate reductase large subunit | 149 | 157 |
| treated | KTMTDTYLL | L | 9 | P32248 | C-C chemokine receptor type 7 | 90 | 98 |
| **Sample** | **Peptide Sequence** | **C-term** | **Length** | **Accession** | **Source Protein** | **Start** | **Stop** |
| treated | KVVDVVRNL | L | 9 | P20591 | Interferon-induced GTP-binding protein Mx1 | 259 | 267 |
| treated | MARKINFLM | M | 9 | P82921 | 28S ribosomal protein S21, mitochondrial | 68 | 76 |
| treated | VSRAHQLVM | M | 9 | P67775 | Serine/threonine-protein phosphatase 2A catalytic subunit alpha isoform | 237 | 245 |
| treated | VTRKSWLWQ | Q | 9 | P82930 | 28S ribosomal protein S34, mitochondrial | 80 | 88 |
| treated | AARKHILVW | W | 9 | Q6P5X5 | UPF0545 protein C22orf39 | 77 | 85 |
| treated | ASAIIIQRW | W | 9 | Q8IZT6 | Abnormal spindle-like microcephaly-associated protein | 2068 | 2076 |
| treated | ASLDISRKW | W | 9 | Q9NWU5 | 39S ribosomal protein L22, mitochondrial | 37 | 45 |
| treated | ATDAAIRVW | W | 9 | Q12788 | Transducin beta-like protein 3 | 172 | 180 |
| treated | FVKKLEHSW | W | 9 | A2A3N6 | Putative PIP5K1A and PSMD4-like protein | 358 | 366 |
| treated | GTHSLDIKW | W | 9 | Q9UHQ1 | Nuclear prelamin A recognition factor | 448 | 456 |
| treated | HAIPLRSSW | W | 9 | P62873 | Guanine nucleotide-binding protein G(I)/G(S)/G(T) subunit beta-1 | 91 | 99 |
| treated | HARPEEPSW | W | 9 | Q9NQ89 | Uncharacterized protein C12orf4 | 113 | 121 |
| treated | HSHPHITVW | W | 9 | P46736 | Lys-63-specific deubiquitinase BRCC36 | 122 | 130 |
| treated | HTMQRQKVW | W | 9 | Q9NWU2 | Protein C20orf11 | 165 | 173 |
| treated | ISKRLGRRW | W | 9 | O15370 | Transcription factor SOX-12 | 70 | 78 |
| treated | ITYDKLNKW | W | 9 | P05120 | Plasminogen activator inhibitor 2 | 286 | 294 |
| treated | ITYQHIDRW | W | 9 | Q9UBQ5 | Eukaryotic translation initiation factor 3 subunit K | 148 | 156 |
| treated | KAMTGVEQW | W | 9 | P49368 | T-complex protein 1 subunit gamma | 427 | 435 |
| treated | KLKPLLEKW | W | 9 | P09086 | POU domain, class 2, transcription factor 2 | 256 | 264 |
| treated | KSGAIIEKW | W | 9 | Q67FW5 | UDP-GlcNAc:betaGal beta-1,3-N-acetylglucosaminyltransferase-like protein 1 | 61 | 69 |
| treated | KSIDLIQKW | W | 9 | P49585 | Choline-phosphate cytidylyltransferase A | 270 | 278 |
| treated | KSIRNDIEW | W | 9 | Q9NPA3 | Mid1-interacting protein 1 | 95 | 103 |
| treated | KSRLSISGW | W | 9 | Q8N543 | 2-oxoglutarate and iron-dependent oxygenase domain-containing protein 1 | 228 | 236 |
| treated | KTKETSVNW | W | 9 | P55265 | Double-stranded RNA-specific adenosine deaminase | 1120 | 1128 |
| treated | KTQGPRALW | W | 9 | Q96AG3 | Solute carrier family 25 member 46 | 152 | 160 |
| treated | KVREEYRKW | W | 9 | P48960 | CD97 antigen | 792 | 800 |
| treated | KVRKWEKKW | W | 9 | Q9BQE9 | B-cell CLL/lymphoma 7 protein family member B | 27 | 35 |
| treated | LAKVIHEKW | W | 9 | Q86UW6 | NEDD4-binding protein 2 | 1415 | 1423 |
| treated | LSRRVEKHW | W | 9 | Q2VIR3 | Putative eukaryotic translation initiation factor 2 subunit 3-like protein | 443 | 451 |
| treated | RAKQFHEAW | W | 9 | Q03001 | Dystonin | 6705 | 6713 |
| treated | RSHDTLVRW | W | 9 | Q9NU22 | Midasin | 177 | 185 |
| treated | RVKTHLPSW | W | 9 | P17026 | Zinc finger protein 22 | 203 | 211 |
| treated | RVTTIARNW | W | 9 | Q15437 | Protein transport protein Sec23B | 497 | 505 |
| treated | SSDKSVKVW | W | 9 | Q9GZS3 | WD repeat-containing protein 61 | 251 | 259 |
| treated | SSDTTVKVW | W | 9 | Q8TAF3 | WD repeat-containing protein 48 | 94 | 102 |
| treated | TAKALQAHW | W | 9 | Q96EZ8 | Microspherule protein 1 | 247 | 255 |
| treated | VSAIVEQSW | W | 9 | P49459 | Ubiquitin-conjugating enzyme E2 A | 141 | 149 |
| treated | VTALAARTW | W | 9 | Q9UII2 | ATPase inhibitor, mitochondrial | 3 | 11 |
| treated | VTRAKQIVW | W | 9 | P00558 | Phosphoglycerate kinase 1 | 328 | 336 |
| treated | VTYKNVPNW | W | 9 | P62826 | GTP-binding nuclear protein Ran | 96 | 104 |
| treated | YAKRAFVHW | W | 9 | Q9BQE3 | Tubulin alpha-1C chain | 399 | 407 |
| **Sample** | **Peptide Sequence** | **C-term** | **Length** | **Accession** | **Source Protein** | **Start** | **Stop** |
| treated | YTHQVVTRW | W | 9 | P50613 | Cyclin-dependent kinase 7 | 169 | 177 |
| treated | HASTLHRLY | Y | 9 | Q14152 | Eukaryotic translation initiation factor 3 subunit A | 292 | 300 |
| treated | KALIYQKKY | Y | 9 | Q99996 | A-kinase anchor protein 9 | 3717 | 3725 |
| treated | RARELLVSY | Y | 9 | Q9Y4W2 | Ribosomal biogenesis protein LAS1L | 268 | 276 |
| treated | RSRIQVWLY | Y | 9 | P62304 | Small nuclear ribonucleoprotein E | 28 | 36 |
| treated | RTAHVILRY | Y | 9 | Q9UKV5 | E3 ubiquitin-protein ligase AMFR | 229 | 237 |
| treated | VSFQHPHKY | Y | 9 | Q8N1B3 | Cyclin-related protein FAM58A | 136 | 144 |
| treated | LSRPDGSASF | F | 10 | Q9NQT4 | Exosome complex component RRP46 | 38 | 47 |
| treated | STAKEIIQHF | F | 10 | Q9UET6 | Putative ribosomal RNA methyltransferase 1 | 96 | 105 |
| treated | VSSRTRSRSL | L | 10 | Q6T4R5 | Nance-Horan syndrome protein | 477 | 486 |
| treated | AAKPEQIQKW | W | 10 | Q99986 | Serine/threonine-protein kinase VRK1 | 90 | 99 |
| treated | ASARAGIHLW | W | 10 | Q9HAY2 | Melanoma-associated antigen F1 | 298 | 307 |
| treated | ASKKLDLEAW | W | 10 | P49591 | Serine--tRNA ligase, cytoplasmic | 373 | 382 |
| treated | ASYQDRRQSW | W | 10 | Q9H410 | Kinetochore-associated protein DSN1 homolog | 87 | 96 |
| treated | GTKKYDLSKW | W | 10 | Q9UM54 | Unconventional myosin-VI | 1080 | 1089 |
| treated | HTKRVGIVAW | W | 10 | P31146 | Coronin-1A | 130 | 139 |
| treated | HTLDDRTQLW | W | 10 | Q9BXP5 | Serrate RNA effector molecule homolog | 529 | 538 |
| treated | HTQDVKHVVW | W | 10 | O76071 | Probable cytosolic iron-sulfur protein assembly protein CIAO1 | 149 | 158 |
| treated | KAREYSKEGW | W | 10 | Q5XKP0 | Protein QIL1 | 101 | 110 |
| treated | KAVDIVKQVW | W | 10 | Q460N5 | Poly [ADP-ribose] polymerase 14 | 726 | 735 |
| treated | KAWRGTLARW | W | 10 | B0I1T2 | Unconventional myosin-Ig | 719 | 728 |
| treated | KSHYDEAYKW | W | 10 | Q92624 | Amyloid protein-binding protein 2 | 219 | 228 |
| treated | KTKRVLPPNW | W | 10 | P62277 | 40S ribosomal protein S13 | 130 | 139 |
| treated | KTLAEINQKW | W | 10 | Q99728 | BRCA1-associated RING domain protein 1 | 209 | 218 |
| treated | KTSELLVRKW | W | 10 | Q9BVM2 | Protein DPCD | 43 | 52 |
| treated | KVGSVVSVGW | W | 10 | Q8WUM9 | Sodium-dependent phosphate transporter 1 | 626 | 635 |
| treated | LSRKQKEELW | W | 10 | Q86XI2 | Condensin-2 complex subunit G2 | 42 | 51 |
| treated | LTRISDPEKW | W | 10 | Q14562 | ATP-dependent RNA helicase DHX8 | 391 | 400 |
| treated | RALELEARRW | W | 10 | Q03169 | Tumor necrosis factor alpha-induced protein 2 | 334 | 343 |
| treated | RSRARAGELW | W | 10 | Q9BXR0 | Queuine tRNA-ribosyltransferase | 25 | 34 |
| treated | RSRDISREEW | W | 10 | Q9ULV3 | Cip1-interacting zinc finger protein | 767 | 776 |
| treated | RSRINEKSVW | W | 10 | Q9Y576 | Ankyrin repeat and SOCS box protein 1 | 63 | 72 |
| treated | RTAGHPLTRW | W | 10 | P35236 | Tyrosine-protein phosphatase non-receptor type 7 | 76 | 85 |
| treated | RTMNIKSATW | W | 10 | Q16665 | Hypoxia-inducible factor 1-alpha | 180 | 189 |
| treated | RVAYQKDRRW | W | 10 | Q9NTJ3 | Structural maintenance of chromosomes protein 4 | 726 | 735 |
| treated | RVINEEYKIW | W | 10 | Q09028 | Histone-binding protein RBBP4 | 15 | 24 |
| treated | RVKSVNLDQW | W | 10 | Q8IYB5 | Stromal membrane-associated protein 1 | 69 | 78 |
| treated | SAKSARLNLW | W | 10 | Q7KZF4 | Staphylococcal nuclease domain-containing protein 1 | 884 | 893 |
| treated | SATDAAIRVW | W | 10 | Q12788 | Transducin beta-like protein 3 | 171 | 180 |
| treated | SSKRAELEKW | W | 10 | Q9UH65 | Switch-associated protein 70 | 458 | 467 |
| treated | TSSSSVRVEW | W | 10 | Q9NZ45 | CDGSH iron-sulfur domain-containing protein 1 | 4 | 13 |
| treated | VSAEGVLHVW | W | 10 | P15260 | Interferon gamma receptor 1 | 215 | 224 |
| **Sample** | **Peptide Sequence** | **C-term** | **Length** | **Accession** | **Source Protein** | **Start** | **Stop** |
| treated | VSKKVSEDGW | W | 10 | Q8TBP6 | Solute carrier family 25 member 40 | 183 | 192 |
| treated | VSQKISKTRW | W | 10 | Q8NFH3 | Nucleoporin Nup43 | 9 | 18 |
| treated | VTKDGRVFAW | W | 10 | P18754 | Regulator of chromosome condensation | 360 | 369 |
| treated | KTLPADVQNY | Y | 10 | Q99590 | Protein SCAF11 | 1152 | 1161 |
| treated | KTLTPIIQEY | Y | 10 | Q53EL6 | Programmed cell death protein 4 | 166 | 175 |
| treated | RSRKELLNFY | Y | 10 | Q9Y3A4 | Ribosomal RNA-processing protein 7 homolog A | 233 | 242 |
| treated | KAVRDALKTEF | F | 11 | P56381 | ATP synthase subunit epsilon, mitochondrial | 21 | 31 |
| treated | KSKDIVNKMTF | F | 11 | P52732 | Kinesin-like protein KIF11 | 757 | 767 |
| treated | KTRANVDKVFF | F | 11 | P11234 | Ras-related protein Ral-B | 160 | 170 |
| treated | KTYNEPGSQVF | F | 11 | Q86U86 | Protein polybromo-1 | 260 | 270 |
| treated | RTLPVDSSHGF | F | 11 | Q6P4F7 | Rho GTPase-activating protein 11A | 328 | 338 |
| treated | RTRPAANPIQF | F | 11 | P23921 | Ribonucleoside-diphosphate reductase large subunit | 740 | 750 |
| treated | KSLSDTAKPHI | I | 11 | Q13118 | Krueppel-like factor 10 | 120 | 130 |
| treated | RTKLQNKEHVI | I | 11 | P27635 | 60S ribosomal protein L10 | 139 | 149 |
| treated | RTLEDRGIRKI | I | 11 | Q9Y2X3 | Nucleolar protein 58 | 396 | 406 |
| treated | KEKQRLLVLSK | K | 11 | P29475 | Nitric oxide synthase, brain | 1125 | 1135 |
| treated | VSSRTVGSRSL | L | 11 | O75159 | Suppressor of cytokine signaling 5 | 167 | 177 |
| treated | VTELVQSKVET | T | 11 | Q9C0G6 | Dynein heavy chain 6, axonemal | 1392 | 1402 |
| treated | HSFNQSSSSQW | W | 11 | Q9Y580 | RNA-binding protein 7 | 179 | 189 |
| treated | HSNEQTLQRSW | W | 11 | Q9Y6J8 | Serine/threonine/tyrosine-interacting-like protein 1 | 263 | 273 |
| treated | HSSEELRNLGW | W | 11 | Q9BYW2 | Histone-lysine N-methyltransferase SETD2 | 1250 | 1260 |
| treated | HSYSPRAIHSW | W | 11 | P28070 | Proteasome subunit beta type-4 | 126 | 136 |
| treated | IVRPSIVGASW | W | 11 | Q8WVX9 | Fatty acyl-CoA reductase 1 | 226 | 236 |
| treated | KAIEEGVNHMW | W | 11 | P46926 | Glucosamine-6-phosphate isomerase 1 | 214 | 224 |
| treated | KAWESSSRQKW | W | 11 | Q14679 | Tubulin polyglutamylase TTLL4 | 708 | 718 |
| treated | KSFIQKKMQNW | W | 11 | Q9BY44 | Eukaryotic translation initiation factor 2A | 121 | 131 |
| treated | KSRFSGEGKNW | W | 11 | Q96RL7 | Vacuolar protein sorting-associated protein 13A | 1748 | 1758 |
| treated | KSVTAHLAAKW | W | 11 | Q9NYU1 | UDP-glucose:glycoprotein glucosyltransferase 2 | 33 | 43 |
| treated | KTKPEGRSVTW | W | 11 | Q8N6M6 | Aminopeptidase O | 249 | 259 |
| treated | LTKEDGEKETW | W | 11 | Q9NUA8 | Zinc finger and BTB domain-containing protein 40 | 670 | 680 |
| treated | LTKQGGLVKTW | W | 11 | Q9UN19 | Dual adapter for phosphotyrosine and 3-phosphotyrosine and 3-phosphoinositide | 171 | 181 |
| treated | QTKAKVTGDEW | W | 11 | P13807 | Glycogen [starch] synthase, muscle | 48 | 58 |
| treated | RALEAEKRALW | W | 11 | Q14160 | Protein scribble homolog | 1491 | 1501 |
| treated | RARLEESRRLW | W | 11 | Q01082 | Spectrin beta chain, brain 1 | 631 | 641 |
| treated | RGTTILAKHAW | W | 11 | P51809 | Vesicle-associated membrane protein 7 | 10 | 20 |
| treated | RTIQTPIGSTW | W | 11 | Q5TAP6 | U3 small nucleolar RNA-associated protein 14 homolog C | 696 | 706 |
| treated | RTRNEELAQTW | W | 11 | Q8TAT6 | Nuclear protein localization protein 4 homolog | 533 | 543 |
| treated | RVLPPSHRVTW | W | 11 | P15498 | Proto-oncogene vav | 15 | 25 |
| treated | TSRPPAQGASW | W | 11 | O14976 | Cyclin-G-associated kinase | 1121 | 1131 |
| treated | VTSIGTAIRYW | W | 11 | O60337 | E3 ubiquitin-protein ligase MARCH6 | 83 | 93 |
| treated | ITKPQNLNDAY | Y | 11 | O60493 | Sorting nexin-3 | 12 | 22 |
| **Sample** | **Peptide Sequence** | **C-term** | **Length** | **Accession** | **Source Protein** | **Start** | **Stop** |
| treated | KAMQSLKSRGY | Y | 11 | P46783 | 40S ribosomal protein S10 | 47 | 57 |
| treated | KTLPETSLPNY | Y | 11 | Q9BYI3 | Hyccin | 17 | 27 |
| treated | KVRESERAFTY | Y | 11 | Q9BYT1 | Solute carrier family 17 member 9 | 146 | 156 |
| treated | ATSGNDGTIRVW | W | 12 | Q15751 | Probable E3 ubiquitin-protein ligase HERC1 | 3444 | 3455 |
| treated | KLADPDEVARRW | W | 12 | P15036 | Protein C-ets-2 | 392 | 403 |
| treated | KSIKQNKEGMGW | W | 12 | Q8TC07 | TBC1 domain family member 15 | 136 | 147 |
| treated | KSRGYVKEQFAW | W | 12 | P46783 | 40S ribosomal protein S10 | 53 | 64 |
| treated | KTKKDNMKYASW | W | 12 | Q15006 | Tetratricopeptide repeat protein 35 | 248 | 259 |
| treated | RTLDEAVGVQKW | W | 12 | Q14667 | UPF0378 protein KIAA0100 | 836 | 847 |
| treated | SQTSSKPDPSQW | W | 12 | O75410 | Transforming acidic coiled-coil-containing protein 1 | 378 | 389 |
| treated | VSSSHDKSLRLW | W | 12 | Q9UNX4 | WD repeat-containing protein 3 | 691 | 702 |
| treated | ASKEAALANQEVW | W | 13 | Q9BT25 | HAUS augmin-like complex subunit 8 | 322 | 334 |
| treated | RTVSAGKGSATSNW | W | 14 | Q86YP4 | Transcriptional repressor p66-alpha | 570 | 583 |
| Untreated | ITREAQMW | W | 8 | Q14094 | Cyclin-I | 20 | 27 |
| Untreated | LSRKDSRW | W | 8 | Q14152 | Eukaryotic translation initiation factor 3 subunit A | 883 | 890 |
| Untreated | MTGRAARW | W | 8 | Q86TG7 | Retrotransposon-derived protein PEG10 | 125 | 132 |
| Untreated | VTRQGALW | W | 8 | Q5SRD1 | Putative mitochondrial import inner membrane translocase subunit Tim23B | 120 | 127 |
| Untreated | HTQKNRQFF | F | 9 | P86790 | Vacuolar fusion protein CCZ1 homolog B | 85 | 93 |
| Untreated | KAIEKNVLF | F | 9 | P10644 | cAMP-dependent protein kinase type I-alpha regulatory subunit | 130 | 138 |
| Untreated | KSSDIAKTF | F | 9 | P13797 | Plastin-3 | 85 | 93 |
| Untreated | KSTDVAKTF | F | 9 | P13796 | Plastin-2 | 82 | 90 |
| Untreated | KVAEVTKKF | F | 9 | P35914 | Hydroxymethylglutaryl-CoA lyase, mitochondrial | 184 | 192 |
| Untreated | RARTIYERF | F | 9 | Q9BZJ0 | Crooked neck-like protein 1 | 361 | 369 |
| Untreated | RSKTVYEGF | F | 9 | Q9NW38 | E3 ubiquitin-protein ligase FANCL | 20 | 28 |
| Untreated | RTKTKPHLF | F | 9 | Q9H307 | Pinin | 234 | 242 |
| Untreated | SSRRKQLTF | F | 9 | Q8WTV1 | THAP domain-containing protein 3 | 15 | 23 |
| Untreated | VSKPDLITF | F | 9 | Q7Z2F6 | Putative protein ZNF720 | 47 | 55 |
| Untreated | VTHSVRIGF | F | 9 | P26010 | Integrin beta-7 | 185 | 193 |
| Untreated | ITKTVVENI | I | 9 | P57740 | Nuclear pore complex protein Nup107 | 635 | 643 |
| Untreated | ISKQFHHQL | L | 9 | Q99613 | Eukaryotic translation initiation factor 3 subunit C | 710 | 718 |
| Untreated | KAYPKRPLL | L | 9 | Q9GZR7 | ATP-dependent RNA helicase DDX24 | 381 | 389 |
| Untreated | RAREYNARL | L | 9 | Q00653 | Nuclear factor NF-kappa-B p100 subunit | 442 | 450 |
| Untreated | RSRTIVHTL | L | 9 | Q9NWU5 | 39S ribosomal protein L22, mitochondrial | 198 | 206 |
| Untreated | AADETLRLW | W | 9 | Q12834 | Cell division cycle protein 20 homolog | 462 | 470 |
| Untreated | ATNRITVTW | W | 9 | Q8N2W9 | E3 SUMO-protein ligase PIAS4 | 254 | 262 |
| Untreated | GSASVNSRW | W | 9 | Q5SW79 | Centrosomal protein of 170 kDa | 1222 | 1230 |
| Untreated | GTLKGHNGW | W | 9 | P63244 | Guanine nucleotide-binding protein subunit beta-2-like 1 | 9 | 17 |
| Untreated | GTLSGHASW | W | 9 | Q9GZS3 | WD repeat-containing protein 61 | 226 | 234 |
| Untreated | GTVLKTSSW | W | 9 | Q460N5 | Poly [ADP-ribose] polymerase 14 | 1074 | 1082 |
| Untreated | GTYGVRAAW | W | 9 | P13598 | Intercellular adhesion molecule 2 | 258 | 266 |
| Untreated | HTNPVGTEW | W | 9 | Q9NV66 | tRNA wybutosine-synthesizing protein 1 homolog | 427 | 435 |
| **Sample** | **Peptide Sequence** | **C-term** | **Length** | **Accession** | **Source Protein** | **Start** | **Stop** |
| Untreated | IAAQTGTRW | W | 9 | Q9BZL1 | Ubiquitin-like protein 5 | 31 | 39 |
| Untreated | KAKVTGDEW | W | 9 | P13807 | Glycogen [starch] synthase, muscle | 50 | 58 |
| Untreated | KLKDIRNAW | W | 9 | Q9UKD2 | mRNA turnover protein 4 homolog | 52 | 60 |
| Untreated | KVASALEKW | W | 9 | Q9UIG0 | Tyrosine-protein kinase BAZ1B | 1144 | 1152 |
| Untreated | KVKEQKDYW | W | 9 | Q13137 | Calcium-binding and coiled-coil domain-containing protein 2 | 197 | 205 |
| Untreated | LAAIRHARW | W | 9 | Q12905 | Interleukin enhancer-binding factor 2 | 202 | 210 |
| Untreated | LAALRHARW | W | 9 | Q9UPR6 | Zinc finger RNA-binding protein 2 | 769 | 777 |
| Untreated | LAAVRGEQW | W | 9 | P13498 | Cytochrome b-245 light chain | 123 | 131 |
| Untreated | LSKSSATLW | W | 9 | Q3KQU3 | MAP7 domain-containing protein 1 | 270 | 278 |
| Untreated | LSQEQLRQW | W | 9 | P32248 | C-C chemokine receptor type 7 | 347 | 355 |
| Untreated | NSRSEAPNW | W | 9 | P60228 | Eukaryotic translation initiation factor 3 subunit E | 429 | 437 |
| Untreated | RAKRQGDIW | W | 9 | Q92616 | Translational activator GCN1 | 131 | 139 |
| Untreated | RMFAPTKTW | W | 9 | P36578 | 60S ribosomal protein L4 | 100 | 108 |
| Untreated | RVAQMKRTW | W | 9 | Q8IY37 | Probable ATP-dependent RNA helicase DHX37 | 835 | 843 |
| Untreated | RVIDVGSEW | W | 9 | Q00403 | Transcription initiation factor IIB | 44 | 52 |
| Untreated | SADGTIKLW | W | 9 | Q12788 | Transducin beta-like protein 3 | 540 | 548 |
| Untreated | SADGTLKLW | W | 9 | O14727 | Apoptotic protease-activating factor 1 | 762 | 770 |
| Untreated | SSSRIRAAW | W | 9 | P14543 | Nidogen-1 | 4 | 12 |
| Untreated | SSSSVRVEW | W | 9 | Q9NZ45 | CDGSH iron-sulfur domain-containing protein 1 | 5 | 13 |
| Untreated | STDRHIRLW | W | 9 | Q9GZL7 | Ribosome biogenesis protein WDR12 | 316 | 324 |
| Untreated | TMRRHEETW | W | 9 | Q5JTW2 | Centrosomal protein of 78 kDa | 204 | 212 |
| Untreated | TSDRHIRIW | W | 9 | Q12834 | Cell division cycle protein 20 homolog | 377 | 385 |
| Untreated | TTHNQSRKW | W | 9 | P47914 | 60S ribosomal protein L29 | 8 | 16 |
| Untreated | VTNKSVSVW | W | 9 | Q9H974 | Queuine tRNA-ribosyltransferase subunit QTRTD1 | 108 | 116 |
| Untreated | VTNPHTDAW | W | 9 | O75293 | Growth arrest and DNA damage-inducible protein GADD45 beta | 122 | 130 |
| Untreated | KSKITHPVY | Y | 9 | Q9UGP8 | Translocation protein SEC63 homolog | 631 | 639 |
| Untreated | KTFPYQHRY | Y | 9 | Q8NI27 | THO complex subunit 2 | 513 | 521 |
| Untreated | KTKFPAEQY | Y | 9 | Q15631 | Translin | 76 | 84 |
| Untreated | KAHPPELKKF | F | 10 | A8MWD9 | Small nuclear ribonucleoprotein G-like protein | 3 | 12 |
| Untreated | KTLHDTRTHF | F | 10 | Q8IZT6 | Abnormal spindle-like microcephaly-associated protein | 1862 | 1871 |
| Untreated | KTRRPDNTAF | F | 10 | Q9NV96 | Cell cycle control protein 50A | 24 | 33 |
| Untreated | KTVEPTGKRF | F | 10 | P10155 | 60 kDa SS-A/Ro ribonucleoprotein | 362 | 371 |
| Untreated | DRVYIHPFHL | L | 10 | P01019 | Angiotensinogen | 34 | 43 |
| Untreated | ASADGTIKLW | W | 10 | Q12788 | Transducin beta-like protein 3 | 539 | 548 |
| Untreated | ASKGREAMEW | W | 10 | Q9Y3B1 | Protein slowmo homolog 2 | 153 | 162 |
| Untreated | AVTALAARTW | W | 10 | Q9UII2 | ATPase inhibitor, mitochondrial | 2 | 11 |
| Untreated | ETKKDHPYTW | W | 10 | Q9Y570 | Protein phosphatase methylesterase 1 | 278 | 287 |
| Untreated | GSADKTVALW | W | 10 | Q16576 | Histone-binding protein RBBP7 | 291 | 300 |
| Untreated | GSSDGTIRLW | W | 10 | Q8TAF3 | WD repeat-containing protein 48 | 228 | 237 |
| Untreated | HTAVVEDVSW | W | 10 | Q09028 | Histone-binding protein RBBP4 | 226 | 235 |
| Untreated | IAANEENRKW | W | 10 | O75368 | SH3 domain-binding glutamic acid-rich-like protein | 39 | 48 |
| Untreated | KARVETQNHW | W | 10 | P28066 | Proteasome subunit alpha type-5 | 91 | 100 |
| **Sample** | **Peptide Sequence** | **C-term** | **Length** | **Accession** | **Source Protein** | **Start** | **Stop** |
| Untreated | KTNIQKEATW | W | 10 | P52292 | Importin subunit alpha-2 | 348 | 357 |
| Untreated | KTQTPNRKEW | W | 10 | P09874 | Poly [ADP-ribose] polymerase 1 | 324 | 333 |
| Untreated | LVSKIGDKNW | W | 10 | Q14008 | Cytoskeleton-associated protein 5 | 865 | 874 |
| Untreated | MTAGVDGHSW | W | 10 | O75694 | Nuclear pore complex protein Nup155 | 450 | 459 |
| Untreated | QTMRRHEETW | W | 10 | Q5JTW2 | Centrosomal protein of 78 kDa | 203 | 212 |
| Untreated | RSVAQAGVQW | W | 10 | Q8N7M2 | Zinc finger protein 283 | 4 | 13 |
| Untreated | SATSSSQRDW | W | 10 | Q96T88 | E3 ubiquitin-protein ligase UHRF1 | 388 | 397 |
| Untreated | SSGDGTLRLW | W | 10 | P57081 | tRNA (guanine-N(7)-)-methyltransferase subunit WDR4 | 207 | 216 |
| Untreated | VTQRKDDSTW | W | 10 | Q9HB58 | Sp110 nuclear body protein | 395 | 404 |
| Untreated | KIYPGHGRRY | Y | 10 | P83731 | 60S ribosomal protein L24 | 12 | 21 |
| Untreated | KTAVIDHHNY | Y | 10 | P39656 | Dolichyl-diphosphooligosaccharide--protein glycosyltransferase 48 kDa subunit | 153 | 162 |
| Untreated | RSRKESYSVY | Y | 10 | Q99880 | Histone H2B type 1-L | 32 | 41 |
| Untreated | KSKPVEKNYAF | F | 11 | P09884 | DNA polymerase alpha catalytic subunit | 431 | 441 |
| Untreated | VASKEIGKRKL | L | 11 | O60293 | Zinc finger C3H1 domain-containing protein | 923 | 933 |
| Untreated | ASYSGKAADVW | W | 11 | Q96RU7 | Tribbles homolog 3 | 235 | 245 |
| Untreated | GTYMGHTGAVW | W | 11 | Q13347 | Eukaryotic translation initiation factor 3 subunit I | 46 | 56 |
| Untreated | GTYSGKAADVW | W | 11 | Q96RU8 | Tribbles homolog 1 | 258 | 268 |
| Untreated | HTIGGSRRAAW | W | 11 | P61313 | 60S ribosomal protein L15 | 182 | 192 |
| Untreated | LSKRNPRQINW | W | 11 | P83731 | 60S ribosomal protein L24 | 41 | 51 |
| Untreated | SASRDKTIIMW | W | 11 | P63244 | Guanine nucleotide-binding protein subunit beta-2-like 1 | 33 | 43 |
| Untreated | VVSPHEDMRTW | W | 11 | P42345 | Serine/threonine-protein kinase mTOR | 1643 | 1653 |
| Untreated | ATRSGQNGNQTW | W | 12 | Q9Y252 | E3 ubiquitin-protein ligase RNF6 | 123 | 134 |
| Untreated | KSHLHQKPGQTW | W | 12 | Q96Q83 | Alpha-ketoglutarate-dependent dioxygenase alkB homolog 3 | 31 | 42 |
| Untreated | PGLARQAPKPRKQ | Q | 13 | P81605 | Dermcidin | 49 | 61 |
| Untreated | PPGASPRKKPRKQ | Q | 13 | Q9H0E3 | Histone deacetylase complex subunit SAP130 | 827 | 839 |
