# Supplementary material for: Acyclovir Has Low but Detectable Influence on HLA-B*57:01 Specificity without Inducing Hypersensitivity
Source: PLoS One. 2015 May 29;10(5):e0124878. doi: 10.1371/journal.pone.0124878 (PMC4449000; doi:10.1371/journal.pone.0124878)
Supplement: S2 Table — (DOCX) [file pone.0124878.s007.docx]

Table S2: Number of peptides with C terminal isoleucine in ACI treated vs. untreated cells

|  | Treated | Untreated |
| --- | --- | --- |
| Number of peptides that have isoleucine at C terminus | 9 | 1 |
| Number of peptides that do not have isoleucine at C terminus | 160 | 94 |

Data does not include peptides that were found in both treated and untreated samples. Analyzed for significance by one-sided Fisher’s exact test (p = 0.0726).
